# Study on landslide susceptibility mapping based on rock–soil characteristic factors

**DOI:** 10.1038/s41598-021-94936-5

**Published:** 2021-07-29

**Authors:** Xianyu Yu, Kaixiang Zhang, Yingxu Song, Weiwei Jiang, Jianguo Zhou

**Affiliations:** 1 School of Civil Engineering, Architecture and Environment, Hubei University of Technology, Wuhan, 430068 People’s Republic of China; 2 China Railway Siyuan Survey and Design Group CO., LTD, Wuhan, 430063 People’s Republic of China; 3 Jiangxi Engineering Laboratory on Radioactive Geoscience and Big Data Technology, East China University of Technology, Nanchang, 330013 People’s Republic of China

**Keywords:** Natural hazards, Environmental sciences

## Abstract

This study introduces four rock–soil characteristics factors, that is, Lithology, Rock Structure, Rock Infiltration, and Rock Weathering, which based on the properties of rock formations, to predict Landslide Susceptibility Mapping (LSM) in Three Gorges Reservoir Area from Zigui to Badong. Logistic regression, artificial neural network, support vector machine is used in LSM modeling. The study consists of three main steps. In the first step, these four factors are combined with the 11 basic factors to form different factor combinations. The second step randomly selects training (70% of the total) and validation (30%) datasets out of grid cells corresponding to landslide and non-landslide locations in the study area. The final step constructs the LSM models to obtain different landslide susceptibility index maps and landslide susceptibility zoning maps. The specific category precision, receiver operating characteristic curve, and 5 other statistical evaluation methods are used for quantitative evaluations. The evaluation results show that, in most cases, the result based on Rock Structure are better than the result obtained by traditional method based on Lithology, have the best performance. To further study the influence of rock–soil characteristic factors on the LSM, these four factors are divided into “Intrinsic attribute factors” and “External participation factors” in accordance with the participation of external factors, to generate the LSMs. The evaluation results show that the result based on Intrinsic attribute factors are better than the result based on External participation factors, indicating the significance of Intrinsic attribute factors in LSM. The method proposed in this study can effectively improve the scientificity, accuracy, and validity of LSM.

## Introduction

**Figure 1:**
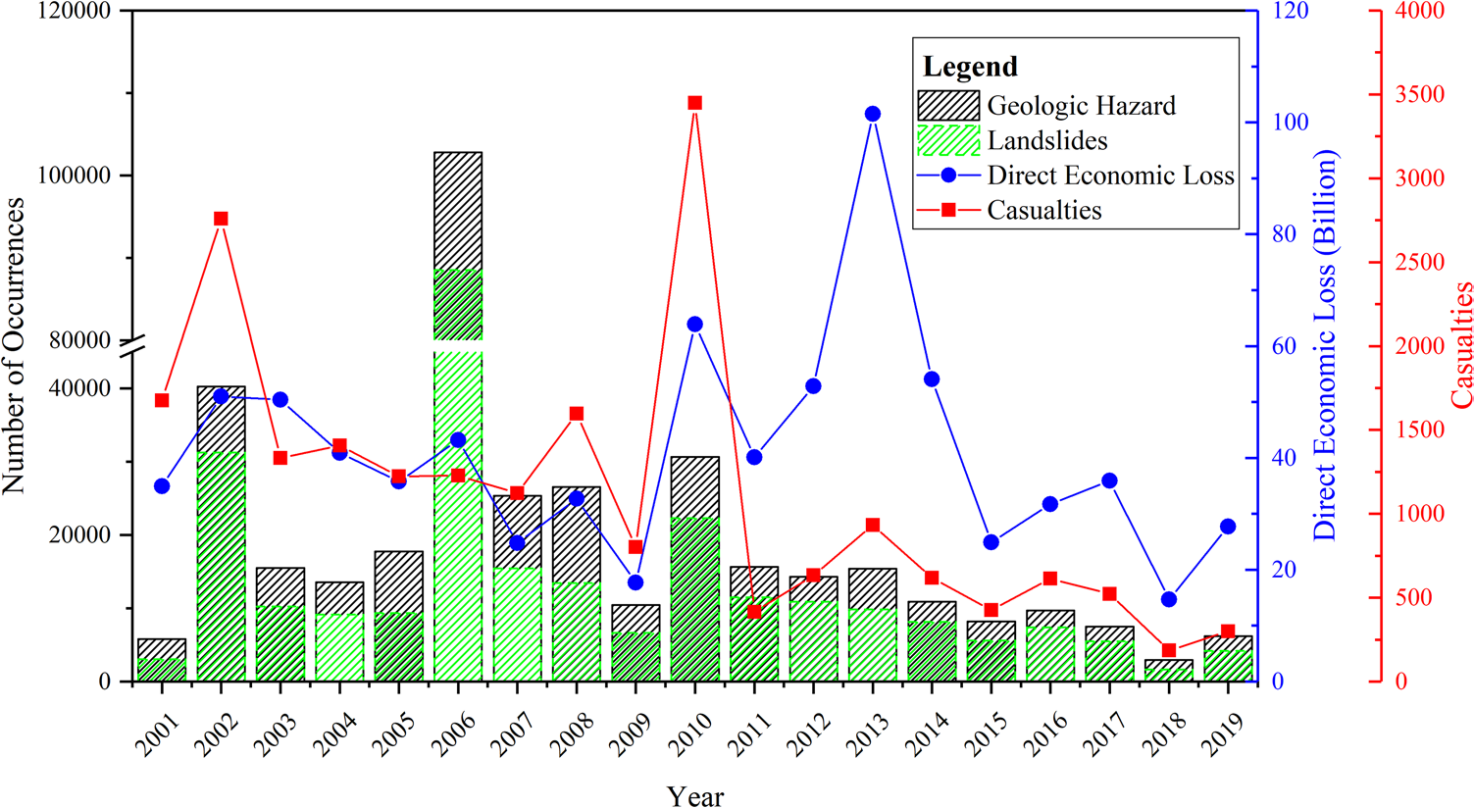
The number of geological disasters and landslides, casualties, and direct economic losses in China from 2001 to 2019^2–4^.

China is located in the eastern part of the Asian continent and has active geotectonic movement and a complex geological environment. Its climate varies from cold temperate to a tropical climate, it is densely populated, and there are extensive and unrestrained engineering activities. It is a country that suffers from frequent landslides^1^. According to data from the National Land and Resources Bulletins and National Geological Hazard Notifications issued by the National Bureau of Statistics of China and the Ministry of Natural Resources of China, there were 379,596 geological hazard events in China during the 19 years from 2001 to 2019, and a total of 21,241 people were injured, missing, or died due to these events. The direct economic loss was as much as RMB 77.87 billion. Among the geological hazard events, there were 274,204 landslides, accounting for 72.2% of the total number of geological hazard events, as shown in Fig. 1.

The Three Gorges Reservoir Area (TGRA) is located in western Hubei and the mountainous areas of Chongqing. It has complex geological and topographic conditions, and suffers frequent occurrences of geological hazards. According to surveys and statistics of the Ministry of Natural Resources, there are over 2000 landslides in the entire TGRA. Among them, there are at least 250 large-scale landslides with total volume of nearly 2.39 billion cubic meters, accounting for 79% of the total number of geological hazard events in the entire TGRA^5^.

The theoretical basis of Landslide Susceptibility Mapping (LSM) is engineering-geological analogy, as a non-deterministic method. It differs from the deterministic method that uses the traditional slope failure mechanics calculation model and predicts the risk of a single landslide combined with basic spatial data^6^. Globally, researchers have conducted many studies on the formation of landslides. With the increasing understanding of landslides, researchers have realized that it is not enough to study the mechanism of single landslide. As landslides feature characteristics of banding and patchy distribution, more attention needs to be paid to research on regional landslides, and LSM is an effective method for the same. With the beginning of the twenty-first century, there has been increasing research on LSMs. Asmelash et al. took the Tamale region on the edge of the rift valley in central Ethiopia as the study area and used Weighted Linear Combination to calculate the Landslide Susceptibility Index (LSI) through the distribution weights and grades given by the analytic hierarchy process. The landslide prediction precision analysis result was about 88.6%^7^. Riegel and Alves applied the Forward Logistic Regression model and the Conditional Analysis model in Sicily, Italy, with the aim to identify the statistical correlation between the spatial distribution of landslides and the past controlling factors. Their studies proved that it was feasible to perform regional LSMs based on factor graphs and landslide records, which can save a lot of time and money^8^. Shahri et al. used the Artificial Neural Network (ANN) to draw a large-scale LSM of southwestern Sweden. The high-precision results obtained using the ANN model were important and cost-effective with potential significance for urban planners^9^. Pandey, Pourghasemi, and Sharma generated the landslide-prone area along Tirpari of Gawal-Himalayas to the Gutu corridor to study the performance of Maximum Entropy and Support Vector Machine (SVM) in LSM. The results of the Receiver Operating Characteristic (ROC) curve showed that SVM had a better prediction rate^10^. Chen et al. set Longhai City in China as the study area and used Priority Decision Tree, Random Forest (RF), and Naive Bayes (NB) models to generate LSMs. The ROC curve results showed that the RF model had the highest accuracy, with an Area Under the Curve (AUC) value of 0.869^11^. Yu et al. considered the spatial correlation of LSM factors, and used the Geographically Weighted Regression model to segment the study area. The LSM was generated with the combination of the Particle Swarm Optimization algorithm and SVM. Compared with the traditional model—not considering the spatial correlation—the results had been significantly improved^6,12^. Sameen, Pradhan, and Lee set the south area of Yangyang City in South Korea as the study area. They used the Bayes Optimization algorithm to obtain the hyperparameters of the One-Dimensional Convolution Neural Network (1D-CNN). And the 1D-CNN was used to generate the LSM of the study area. The results showed that the performance of the 1D-CNN model was better than ANN and SVM, its AUC and prediction precision were the most accurate at 0.893 and 83.11%, respectively^13^. Fang et al. set Yongxin County, Ji’an City, Jiangxi Province as the study area and generated LSMs integrating CNN with three traditional machine learning classifiers, namely, SVM, RF, and Logistic Regression (LR). The experimental results showed that integrating CNN can effectively improve the performance of machine learning classifiers. As for the precision of LSMs, the proposed three methods showed significant improvement compared with traditional machine learning classifiers^14^. Nhu et al. set the Halong region in Vietnam as the study area and studied the ability of Keras’s Deep Learning (DL) model to model the landslide hazard space, and compared it with traditional methods such as RF, J48 Decision Tree, Classification Tree, and Logical Model Tree. The results showed that the prediction precision of Keras’s DL model was 84.0%, indicating that its performance was better than those of traditional methods^15^. The LSM theory has been consistently developing, and has experienced a process from qualitative research to semi-qualitative research, and then to quantitative research^1^. In addition, with the continuous improvement of data acquisition methods and the sharing degree, research on LSM has led to the introduction of the DL model that requires massive amounts of data and complex calculations, greatly promoting the development of LSM theories and applications. Realizing the importance of models for LSM, many researchers devote themselves to the research of LSM models and developed many LSM models with higher accuracy, which often implies that the model is overfitting or the it takes a lot of time to adjust the model parameters. However, few researchers have focused on the underlying elements of landslide events, namely various LSM factors.

Recent years, most researchers continue to focus on rainfall-induced and earthquake-induced landslides in research on LSM factors^16–22^. Moreover, many researchers have focused on other types of LSM factors and explored the influence of LSM factors on LSMs. Lee et al. used web-based digital aerial photographs to generate the LSM in the Jinbu area of South Korea. According to the characteristics of landslides, Lee considered factors related to landslides, such as geomorphology, soil, forest, geology, and land use. The type, average diameter, density, age of trees on the landslide mass were also used as input factors. The RF, Weights of Evidence, LR, and ANN models were used to generate the LSMs. Their overall satisfaction levels were over 80%^23^. Chen, Pourghasemi, and Naghibi studied the influences of 12 factors on the landslides in the study area. They used the precision measurement of the RF model and realized that the most important factors in the study area were lithology, fault distance, and altitude. In the Gini measurement method, the three most important factors were altitude, fault distance, and distance to highway^24^. Pawluszek and Borkowski took the Rhone Lake region in Poland as the study area and studied the Digital Elevation Model (DEM) and the terrain conditions calculated by DEM to discuss the influence of DEM-derived factors on LSMs^25^. Skilodimou et al. used the mountainous area in the northern Peloponnese in Southern Greece as the study area and applied the statistical analysis of landslide frequency and density to evaluate the collected data along with Geographic Information System (GIS), thus determining the influence of natural and human factors on landslide activities^26^. Al-Najjar et al. extracted 14 LSM factors in the study area and divided them into 4 groups based on different methods. Then, they used three machine learning algorithms—RF, NB, and Enhanced Logistic Regression to generate the LSMs. The research results showed that the fourth group with 8 factors selected by factor analysis and optimization methods had the highest AUC value^27^. Yu and Gao used GIS and Remote Sensing (RS) theories to extract 58 LSM factors in the study area. By combining Pearson Correlation Coefficient (PCC), the Principal Component Analysis, and the factor importance analysis, a total of 18 LSM factors were obtained. They also generated the LSM of the TGRA^6^. Mind’je et al. combined 10 factors without multicollinearity with the Frequency Ratio method to generate the LSM of Rwanda. After analysis, it was concluded that parts of the western, northern, and southern regions were the most susceptible areas for landslides and the altitude was the main influencing factor^28^. Bourenane et al. studied the landslide disaster in Azazga city, the statistical results show that the landslides in this area are affected by the dip of the flysch formation layers, the schistosity planes and fractures downward slope direction, and the interface contact between the quaternary scree and flysch substratum^29^. Tang et al. argued that the differences of different types of landslides should be considered when mapping landslide susceptibility, and used loess landslides as a research object. The results of the study showed that rainfall and land use are the keys to predict the occurrence of loess landslides and avalanches^30^. Huang et al. discussed the influence of different attribute intervals (AINs) numbers on the frequency ratio (FR) analysis of continuous environmental factors and the uncertainty of landslide susceptibility prediction (LSP), the results showed that for a certain model, the LSP accuracy gradually increases with the AINs increasing from 4 to 8, and then the accuracy is stable with the AINs increasing from 8 to 20^31^. Huang et al. used machine learning methods such as C5.0 decision tree, LR, multilayer perceptron, and SVM to study the effect of soil erosion (SE) on landslide events in Ningdu County, China. The results show that the SE-based model has higher prediction accuracy than a single model without SE factors^32^. Although many scholars have tried to expand the scope of LSM factors and explore the relationship between factors and LSMs without being limited by traditional influences of rainfall and earthquakes on LSMs, they have achieved different results. These factors and corresponding influences are often concentrated on the influencing factors—the internal conditions formed by landslides are considered as controlling factors, and the external conditions formed by landslides are considered as influencing factors—and there is a lack of discussion on controlling factors.

The purpose of this study is to discuss the influence of rock–soil characteristics factors on LSM, and take the TGRA as the study area. We try to explore the influence of rock–soil characteristics factors and their specific combinations on LSM. We use several statistical methods to evaluate the effectiveness of LSM based on rock–soil characteristics factors, which provides meaningful information for further research.

## Study area and data source

### Overview of study area

**Figure 2:**
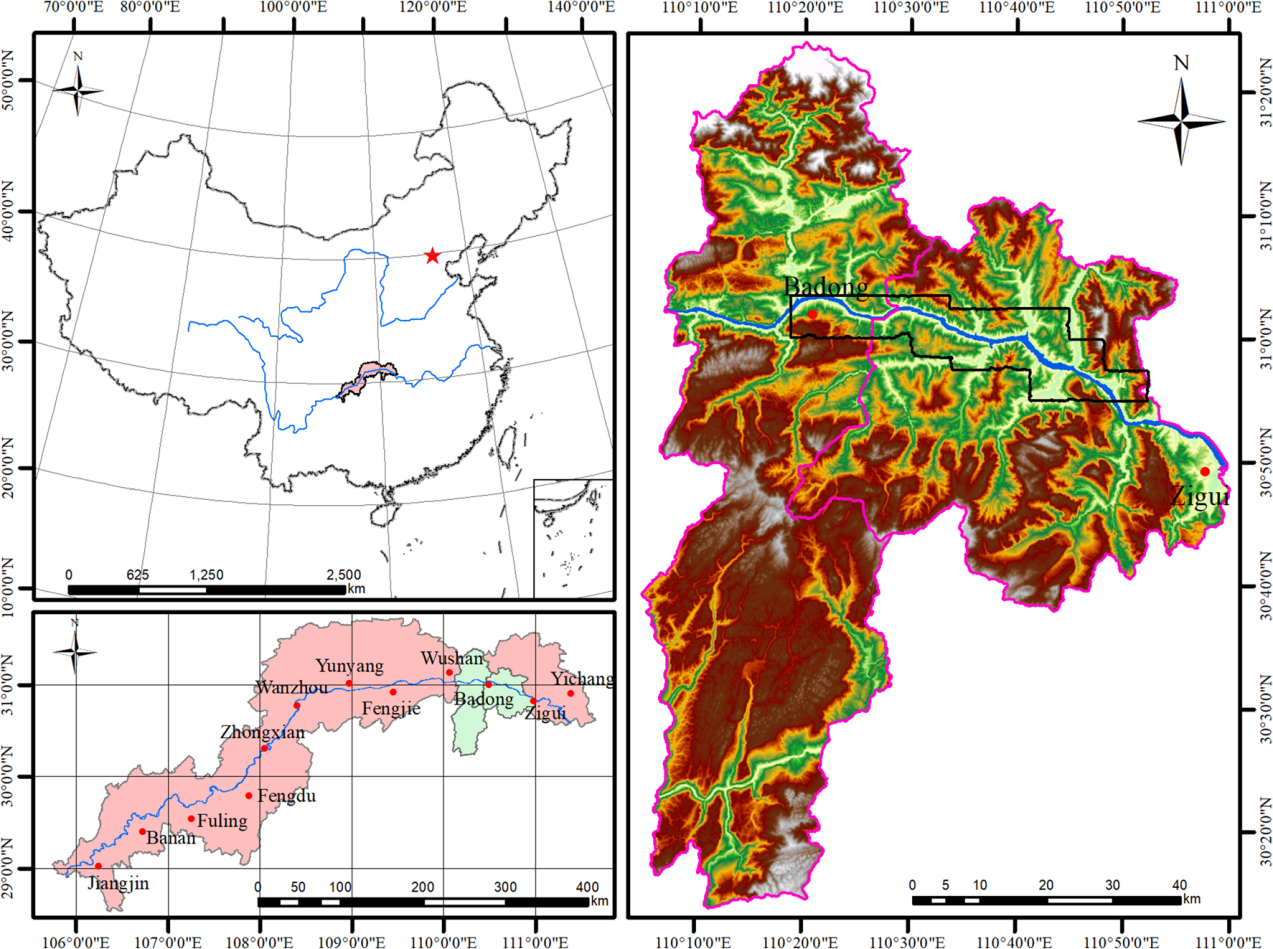
The location of the study area (Drawn with ArcGIS 10.8 software, and the URL is: https://www.esri.com/en-us/arcgis/about-arcgis/overview).

**Figure 3:**
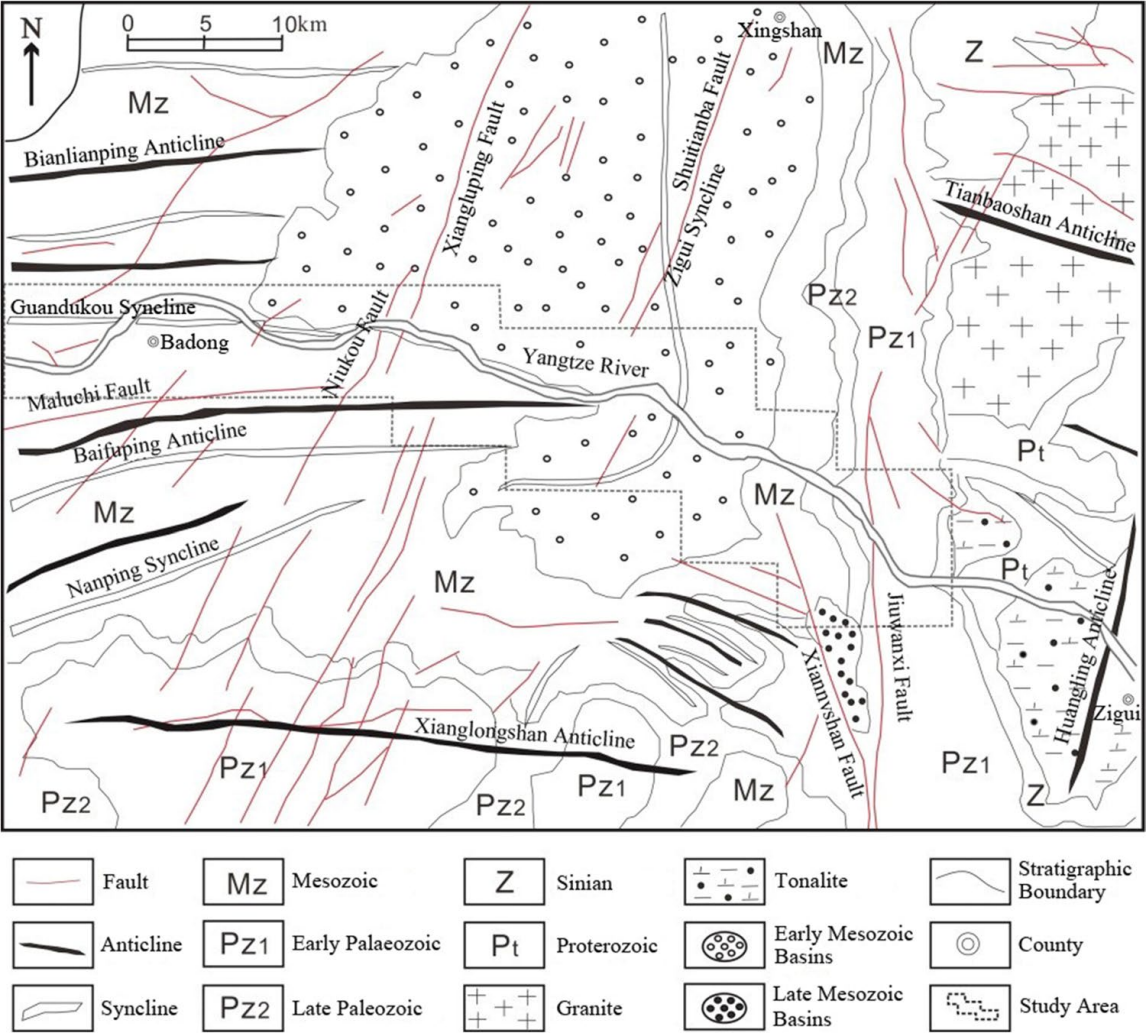
Geological map of the study area (Drawn with ArcGIS 10.8 software, and the URL is: https://www.esri.com/en-us/arcgis/about-arcgis/overview).

The TGRA is located in the transition area from the second step to the third step among the three major steps of Chinese terrain. The study area is in the eastern part of the two natural geographic units of the TGRA, starting from Xinling Town in Badong County and ending at Quyuan Town in Zigui County. It spans about 54 km from east to west and about 16 km from north to south^33^, as shown in Fig. 2. The strata in the study area are well developed, with outcrops from Sinian to Quaternary, and only a few stratigraphic gaps^34^. The structural features in the study area—folds and faults—are mainly formed from the late Yanshan movement to the early Himalayan movement, they form the basic structural background for the evolution and development of the TGRA, and include the Huangling anticline, Zigui syncline, Xiannv Mountain fault, Jiuwanxi fault, Niukou fault, and Xiangluping fault^35^, as shown in Fig. 3. The study area has a well-developed water system, and the density of rivers is as high as 1.2 km per square kilometer^6^. The study area belongs to the mid-latitude subtropical monsoon climate zone, affected by the alternate control of tropical ocean air masses and polar continental air masses. The temperature and rainfall vary significantly with seasons. The average annual rainfall in Badong is 1093 mm, while the average annual rainfall in Zigui is 1274 mm^36^. Since the study area is mostly mountainous, the vegetation (including arable land, shrubs, and woodland, etc.) is lush and occupies the largest area of 258.8 km^2^, accounting for 52.1% of the total area. The water system is well developed, with an area of 120.2 km^2^, accounting for 24.2%. The area of wasteland is affected by the seasons, with a smaller area of 24.1 km^2^, accounting for 18.9%. And the artificial impervious surface (including houses, roads, and bridges, etc.) are mainly concentrated in the area located in the northwest of the study area in Badong County and in the southeast near Zigui County, with an area of 24.1 km^2^, accounting for 4.8%.

### Landslide inventory mapping

**Figure 4:**
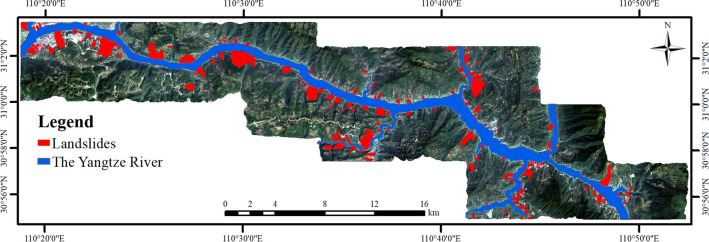
The landslide distribution of the study area (drawn with ArcGIS 10.8 software, and the URL is: https://www.esri.com/en-us/arcgis/about-arcgis/overview).

The existing landslide database shows the spatial distribution of landslide events in the study area, which is also helpful to understand the relationship between LSM factor and landslide occurrence^37^. TGRA landslide inventory map is produced through extensive field investigation, landslide history and bibliographic data on landslides, and visual interpretation of remote sensing images. In this study, 202 historical landslide locations were extracted from the study area, and the distribution is shown in Fig. 4.

Landslide disasters occurred frequently in the study area, especially after the establishment of TGRA. Affected by the reservoir water storage and rainfall, some ancient landslides revived and new landslide disaster were produced. The total area of landslides in the study area is 23.4 km^2^, of which the largest is Fanjiaping landslide with an area of 1.51 km^2^, and the smallest is Lianhua Street landslide with an area of 2068.8 m^2^.

It can be seen from Fig. 4 that the landslide disasters in the study area are distributed along the Yangtze River, showing the characteristics of banding and patchy distribution, and there are compound landslides formed by the superposition of multiple landslides, such as Taijiaozi, Shizishubao, Tanjiawan, Huanglashi, Hualianshu, Zhangjialiangzi landslide group on the left bank of the Yangtze River.

### Data

The data sources used in this paper are shown in Table 1.

**Table 1:** The data source used.

Name	Describe	Data source	Spatial resolution/Scale
Remote sensing data	Landsat-8 operational land imager (OLI +) sensor data, acquired on 15 September 2013, with a path/row number of 127/39	https://earthexplorer.usgs.gov/	30 m
DEM data	Advanced Spaceborne Thermal Emission and Reflection Radiometer Global Digital Elevation Model (ASTER GDEM)	https://lpdaac.usgs.gov/tools/data-pool/	30 m
Basic geographic data	Topographic maps of the study area	Hubei Geological Survey Institute^69^	1:50,000
Basic geological data	Geological maps of the study area	Hubei Geological Survey Institute^69^	1:50,000
The Landslide Distribution Data	Landslide distribution maps of the study area	Geological disaster prevention and control headquarters in the Three Gorges Reservoir Area^70^	1:10,000

The scale of topographic maps and geological maps are 1:50,000, the scale of landslide distribution maps is 1:10,000, and fully matches the precision requirements of RS data sources and DEM data sources with a spatial resolution of 30 m. In order to ensure that data of different scales/resolutions can be used properly in this study, without being affected by the data of different scales/resolutions on the modeling process, the lowest precision of the available data, i.e., Landsat 8 and DEM data with 30-m resolution, was taken as the research precision. While other data, such as geological maps, topographic maps, and landslide maps, are higher than the research precision, they are resampled to 30-m resolution data.

## Methods

### Factor analysis model

#### PCC analysis

In the field of statistics, the PCC also known as the Pearson Product-Moment Correlation Coefficient (PPMCC), which was proposed by Karl Pearson^38^. It is a common method used to measure the degree of linear correlation between two variables^39^, as shown in Formula 1.

1 $$\rho_{X,Y} = \frac{{cov\left( {X,Y} \right)}}{{\sigma_{X} \sigma_{Y} }} = \frac{{E\left[ {\left( {X - \mu_{X} } \right)\left( {Y - \mu_{Y} } \right)} \right]}}{{\sigma_{X} \sigma_{Y} }} = \frac{{E\left( {XY} \right) - E\left( X \right)E\left( Y \right)}}{{\sqrt {E\left( {X^{2} } \right) - E^{2} \left( X \right)} \sqrt {E\left( {Y^{2} } \right) - E^{2} \left( Y \right)} }}$$

where *cov* refers to the covariance, and *E* refers to the mathematical expectation, *X*, *Y* are the individual sample points, *μ*_*X*_, *μ*_*Y*_ are the sample means, *σ*_*X*_, *σ*_*Y*_ are the sample standard deviations.

The value of PCC is between − 1 and 1. A positive value represents a positive correlation, and a negative value represents a negative correlation^40^. The larger the value, the greater the correlation, and vice versa^41^. In LSM, it is necessary to remove LSM factors with strong correlations (with the value > 0.7) to ensure that the selected factors are relatively independent^39^.

#### Multicollinearity analysis

Multicollinearity analysis is one of the methods to evaluate the correlation between LSM factors. Multicollinearity means that the characteristics of at least two predictive features exhibit a high degree of linear correlation in the multiple regression^42^. Variance expansion factor (VIF) and tolerance (TOL) can be used to objectively calculate multicollinearity results, where VIF and TOL are reciprocal to each other. In the case of LSM, if the VIF coefficient of a factor is greater than 10 or the TOL coefficient is less than 0.1, the corresponding factor can be considered to have multicollinearity and should be excluded from the subsequent modeling process^43^.

#### Relief-F analysis

The Relief-F method evaluates the LSM factor value by calculating the correlation between the LSM factor and the landslide to determine the relative importance of the factor to the occurrence of the landslide^44^. The Relief-F algorithm will randomly select a sample *R* from the training set *D*, and construct sample sets *H* and *M* by using k-nearest neighbor samples with a sample label of *R* and different labels from *R*, respectively^45^. For factor set *A*, the weight of the *i*th factor is calculated by Formula 2.

2 $$w_{i} = w_{i} - \sum\limits_{j = 1}^{k} {\frac{{diff\left( {A_{i} ,R,H_{i} } \right)}}{mk}} + \sum\limits_{C \ne Class\left( R \right)} {\left\{ {\frac{p\left( C \right)}{{1 - p\left[ {Class\left( R \right)} \right]}}\sum\limits_{j = 1}^{k} {\frac{{diff\left[ {A_{i} ,R,M_{j} \left( C \right)} \right]}}{mk}} } \right\}}$$

where *C* is the sample label, *p*(*C*) is the probability of class *C*, *Class* (*R*) is the sample label of class *R*, *M*_*j*_(*C*) is the *j*th sample of class *C*, the *diff* (*A*_*i*_, *R*, *H*_*i*_) and *diff* (*A*_*i*_, *R*, *M*_*i*_(*C*)) are distance functions, and the factor importance will be calculated after repeating this process *m* times.

### Classifiers

#### LR model

LR is a multivariate statistical method, which uses logic function to model binary dependent variables^46^. The principle of LR is to transform each LSM factor into a logical variable, and then use the maximum likelihood estimation to obtain the probability value of each factor for the occurrence of the landslide events^47^. The output prediction of LR is defined as Formula 3^48^.

3 $$p = \frac{1}{{1 + e^{ - z} }}$$

where *p* is the probability and *z* is the linear combination of variables, as show in Formula 4.

4 $$z = \beta_{0} + \beta_{1} X_{1} + \beta_{2} X_{2} + \cdots + \beta_{n} X_{n}$$

where (*β*_0_, *β*_1_, *β*_2_, …, *β*_*n*_) and (*X*_1_, *X*_2_, …, *X*_*n*_) are the regression variables and the explanatory variables, respectively.

#### ANN model

**Figure 5:**
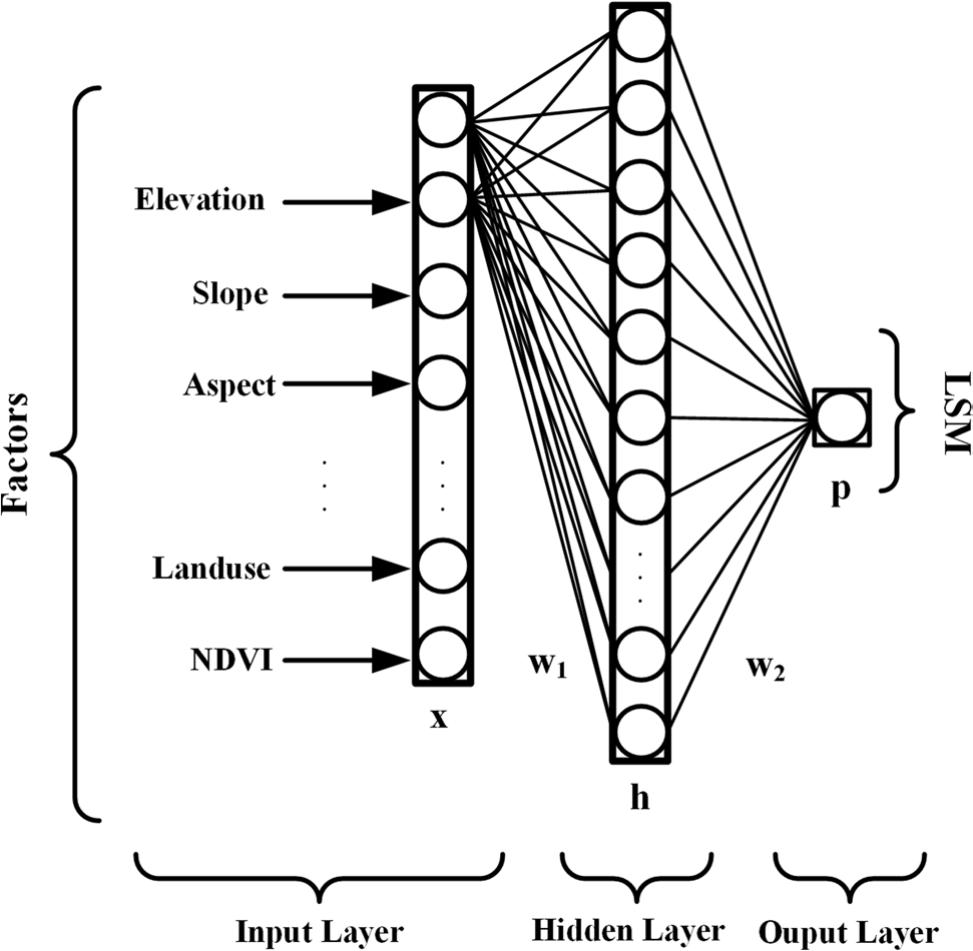
ANN model architecture.

ANN is a computational program that simulates the work of the human brain^49^. The goal of the ANN model is to create a method for predicting the output of input factors that are not used in the modeling process^50^. The standard ANN model consists of three layers: an input layer (i.e., LSM factor), a hidden layer, and an output layer (i.e., LSM). In the training step, the network uses the weights and bias values of the samples to predict the labels of each sample, the cost function finds the difference between the computed labels and the true labels, while in the backpropagation step, each weight receives an update based on the gradient of the cost function, and this process continues until the convergence condition is met or the maximum number of epochs is reached in training^50,51^. A sketch of the ANN architecture is shown in Fig. 5.

#### SVM model

The SVM model was first proposed by Vapnik (1995) as a machine learning algorithm, and was established based on the VC dimension theory and the principle of minimum structural risk^52^. It seeks the best compromise between the complexity of the model and the learning ability based on limited sample information, to obtain the best generalization ability. It have many unique advantages in small sample, nonlinear, and high-dimensional pattern recognitions^52,53^. Due to the relatively small number of landslide samples and the large number of LSM factors in the study area, the SVM model is used as the LSM model in this paper.

SVM can be a binary classification model. In order to find an *n*-dimensional hyperplane, this model divides the statistical samples to ensure the distance between the sample point closest to the hyperplane and the dividing line is the largest^53^. In other words, it is a space classifier that maximizes the interval between sample points. Its function is defined as show in Formula 5.

5 $$\left\{ {\begin{array}{*{20}l} {min\frac{1}{2}\left\| w \right\|^{2} ,} \hfill \\ {s.t.,y_{i} \left( {\left( {w \cdot x_{i} } \right) + b} \right) \ge 1,} \hfill \\ \end{array} } \right.$$

where *x*_*i*_ refers to the point on the hyperplane, *y*_*i*_ refers to the classification mark, *i* = 1, 2, …, *R*, *R* refers to the number of samples, *w* refers to the vector perpendicular to the hyperplane, b refers to the constant to prevent the hyperplane from passing through the origin of the coordinate axis, ||*w*|| is 2-norm of *w*.

Formula 6 introduces a non-negative slack variable *ζ*_*i*_, however, a penalty factor *C* must also be introduced to represent the distance from a misclassified point to its correct position. Therefore, Formula 6 can be expressed as:

6 $$\left\{ {\begin{array}{*{20}l} min\frac{1}{2}\left\| w \right\|^{2} + C\sum\limits_{i = 1}^{n} {\xi_{i} } , \hfill \\ s.t.,y_{i} \left( {\left( {w \cdot x_{i} } \right) + b} \right) \ge 1 - \xi_{i} \hfill \\ \end{array} } \right.$$

For the problem of transforming training samples into *n*-dimensional space, Vapnik considered *K* (*x*_*i*_, *y*_*i*_) as a kernel function and introduced SVM. The essence of this kernel function is a mapping function. Its basic function is to accept vectors in the low-dimensional space and calculate the inner product value of vectors in the *n*-dimensional space after a certain transformation, that is, it can map low-dimensional inseparable linear training samples to *n*-dimensional space and make them linearly separable^52^. In this paper, the Radial Basis Function is selected as the kernel function of the SVM model to map vectors in the low-dimensional space to the high-dimensional feature space for classification. The function can be expressed as Formula 7.

7 $$K\left( {x_{i} ,x_{j} } \right) = \exp \left( { - \gamma \left\| {x_{i} - x_{j} } \right\|^{2} } \right)$$

where *γ* refers to the nuclear parameters of different radial basis functions.

### Result evaluation model

#### Specific category precision analysis

The traditional quantitative analysis method of LSM is based on Landslide Susceptibility Zoning (LSZ), which calculates proportion of the area of the landslide in each type of landslide-prone zones using the landslide distribution data. The analysis result is based on the proportion of the landslide area in the highest susceptibility zones to the total area of the landslides. However, when the prediction results of the model are polarized, and many areas in the LSZ belong to the highest susceptibility zone, it is natural that most landslides are in the highest susceptibility zone, which will lead to a better result of the model. Obviously, this cannot be used to verify the effect of the method, and it is not appropriate to the quantitative analysis of LSM.

The specific category precision analysis method is an improved quantitative analysis method that was used to solve the above problems^12^. In this paper, the specific category precision method takes into account the number of calculation units in the classification area, and can be expressed as Formula 8:

8 $$p_{i} = \frac{{A_{i} }}{{B_{i} }} \cdot 100\%$$

where *i* = 1, 2, …, *S*, *S* refers to the number of LSZ classification, *A*_*i*_ is the number of calculation units occupied by landslides in the *i*th LSZ classification, *B*_*i*_ is the number of calculation units in the *i*th LSZ classification, and *p*_*i*_ is the specific category precision in the *i*th LSZ classification.

#### ROC curve and AUC value

The ROC curve analysis is a classic method in statistical theory and is also a method commonly used to analyze LSM^54^. This method mainly analyzes the classification results of the binary classification model^55^. The ROC curve is in the form of coordinates on a rectangular coordinate system, describes the process of classifier performance as the classifier threshold changes, with a value range of [0,1]. Each point on the curve reflects the sensitivity to the same signal. The horizontal axis is the specificity of the False Positive Rate (FPR), the vertical axis is the sensitivity of the True Positive Rate (TPR). There are 4 situations: (1) the result is a positive type and the prediction is also positive, it is a True Positive (TP); (2) the result is a negative type and the prediction is positive, it is a False Positive (FP); (3) the result is a negative type and the prediction is also negative, it is a True Negative (TN); and (4) the result is a positive type and the prediction is negative, it is a False Negative (FN)^6^, as shown in Table 2.

**Table 2:** Classification theory matrix of ROC curve.

	Prediction		Total
	Positive (P)	Negative (N)	
**True**			
Positive (P)	True Positive, TP	False Negative, FN	Actual Positive, TP + FN
Negative (N)	False Positive, FP	True Negative, TN	Actual Negative, FP + TN
Total	Predicted Positive, TP + FP	Predicted Negative, FN + TN	TP + FP + FN + TN

The AUC value is calculated by the ROC curve. This indicator is widely used in studies in different disciplines and has been tested in various precision prediction models. The prediction effect has also been widely recognized.

#### Five statistical measures

In addition to the specific category accuracy analysis, ROC curve analysis and AUC value mentioned above, five statistical methods, including overall accuracy (OA), precision, recall, F-measure, and Matthews correlation coefficient (MCC), were used to evaluate the calculation results of the model^14^. The formulas of these 5 evaluation methods are as Formula 9–13.

9 $$OA = \frac{TP + TN}{{TP + FP + TN + FN}}$$

10 $$Precision = \frac{TP}{{TP + FP}}$$

11 $$Recall = \frac{TP}{{TP + FN}}$$

12 $$F - measure = \frac{2 \times Presion \times Recall}{{Presion + Recall}}$$

13 $$MCC = \frac{TP \times TN - FP \times FN}{{\sqrt {\left( {TP + FP} \right)\left( {TP + FN} \right)\left( {TN + FP} \right)\left( {TN + FN} \right)} }}$$

where the TP, FP, TN, and FN are the same as the definitions in “ROC curve and AUC value” section.

## Experimental process

### Selection of calculation units

According to the conclusions of Guzzetti et al., all LSM calculation units were summarized into the following five types: grid unit, geographic unit, single condition unit, slope unit, and sub-basin unit^56^. Based on the research purpose, the grid unit is selected as the calculation unit in this paper. After invalid data is extracted and eliminated using ArcGIS 10.5 developed by ESRI, a total of 422,242 valid calculation units in study area are finally obtained.

### Selection of factors

#### Selection of basic factors

Based on previous research results, eleven factors in the study area are selected in this paper, including Elevation, Slope, Aspect, Slope Form, Slope Structures, Distance from River, Topographic Wetness Index (TWI), Stream Power Index (SPI), Rainfall, Landuse, and Normalized Difference Vegetation Index (NDVI). These eleven factors can be divided into two major categories, that is, landslide controlling factors and influencing factors, and four sub-categories, that is, topography, basic geology, hydrological conditions, and land cover. The effect of each factor on landslides is shown in Table 3.

**Table 3:** Basic factors and effect on landslides.

Category	Subcategory	Factor	Effect on landslides
Controlling factor	Topography	Elevation	The spatial distribution of landslides varies with different elevation values and is mainly reflected in the following aspects: vegetation coverage, vegetation type, land use intensity, and rock–soil mass distribution at the critical surface of landslides^57^
		Slope	The slope has great influence on the stress distribution of rock–soil mass on slopes, the surface water runoff on slopes, the recharge and discharge of groundwater in slopes, the thickness of the weathered layer on slopes, the vegetation coverage, and the land use. It can affect the stability of landslides^58^
		Aspect	Different slope directions lead to different intensity of solar radiation and weathering, which affect factors such as the vegetation coverage, water evaporation, and soil humidity. Consequently, the distribution of the groundwater pore pressure of rock–soil mass, as well as the physical and mechanical characteristics change, thus indirectly affecting the slope stability^59^
		Slope form	The slope form refers to the comprehensive index of the plane curvature and the profile curvature, which determines the degree of cutting and fracture of the ground^60^
	Geology	Slope structures	The slope structures type is one of the controlling factors of the formation of geological hazards. Slopes can be divided in accordance with the spatial relationship between the four parameters, including slope, aspect, inclination, and tendency of the underlying stratum of the slope. The nature, characteristics, and degree of development of geological hazards are different on various slope structures^61^
	Hydrological	Distance from river	Due to the immersion of river, the rock–soil mass near the river is prone to softening. And, the underlying rock layer immersed in the river is hollowed out by the water flow, causing suspension of the landslide mass. Therefore, water flow has adverse effects on the stability of the slope rock–soil mass. Landslide hazards may be more likely to occur in areas closer to water^59^
		Topographic Wetness Index	The Topographic Wetness Index (TWI) is an important factor for LSM as it indicates the conditions of soil, geography, and runoff volume^62^
		Stream Power Index	The Stream Power Index (SPI) indicates the erosion capacity of a stream. In general, landslides are more likely to occur in areas that have experienced severe erosion^62^
Influencing factor	Atmospheric precipitation	Rainfall	Rainfall can infiltrate along cracks in landslide mass, severely affecting the shear strength of slopes, and important stages in the evolution of slope morphology caused by shallow landslides are usually associated with short but intense rainfall events^14,63^
	Human engineering activities	Landuse	As it is closely related to human engineering activities, landuse is very important in slope stability research, and therefore, it has been widely used in landslide modeling^64^
	Vegetation Index	Normalized Difference Vegetation Index	Vegetation can improve the shear strength of the soil and fix the soil through the interaction between the root system and the soil. It can also reduce soil erosion and maintain rock–soil mass stability. Therefore, vegetation has an important effect on the stability of rock–soil mass on slopes^9^

#### Rock–soil characteristic factors

Rock–soil mass is the material basis for landslides. Rock–soil mass with different characteristics has diverse effects on the development of landslides. It not only affects the development degree of landslides in the study area, but also determines the type and scale of landslides. It is an important controlling factor for landslides^1^. The structure and composition of rock–soil mass in the study area constitute unique rock–soil characteristic factors. Based on the characteristics of the rock–soil mass in the study area, the properties of rock–soil mass are summarized into four rock–soil characteristic factors: lithology, Rock Structure, rock infiltration, and rock weathering. The effects of each rock–soil characteristic factor on landslides are shown in Table 4.

**Table 4:** Rock–soil characteristic factors and effect on landslides.

Category	Subcategory	Factor	Effect on landslides
Controlling factor	Geology	Lithology	Based on statistical analysis, there is a close relationship between the spatial distribution characteristics of landslides and the rock hardness in the study area. The slope deformation and failure effect are relatively weak in areas where the rock hardness is strong. In such a case, landslides do not easily develop. Large and medium-sized landslides are commonly found in slopes with a moderate hardness. In areas where the rock hardness is weak, the slope deformation and failure effect are intensified, and the area is prone to landslides^14^
		Rock structure	The rock mass structure is an internal characteristic of rock mass, with determined by the shape, scale, nature, combination, and connection characteristics of the rock–soil mass structure surface and the structure. The distribution of the landslide area in the study area is highly correlated with the rock mass structure
		Rock infiltration	Rock infiltration refers to the flowability of fluids in rock crevices under gravity. Rock infiltration is closely related to the distribution of landslides in the study area. Rainfall and river water can enter the crevices between rock–soil mass, reducing the shear strength of rock–soil mass and increasing the pressure difference between internal and external, thus inducing landslides
		Rock weathering	Rock weathering refers to the breaking, loosening, and changes in mineral composition of rock–soil mass under the action of solar radiation, light, sunshine, air, water, and organisms. It can destroy the structure of rock–soil mass and result in the overall loosening. Moreover, it can oxidize the surface of rock–soil mass, increase the porosity between rocks, leading to the rock–soil mass more loose, swell, and fragile

In the National Standards of the People's Republic of China, the classification standards for these four factors are specified in detail^65,66^. Therefore, combining the existing lithological data of the study area, the distribution map of these four geotechnical characteristics can be obtained.

For example, in the lithological factors, hard rocks represented by the Huanglong Formation, soft rocks represented by the Liantuo Formation, and soft-hard alternating rocks represented by the Qianfuya Formation. In the rock structure factors, massive structure represented by the Maokou Formation, stratified structure represented by the Liangshan Formation, cataclastic structure represented by the Tongzhuyuan Formation, and granular structure represented by the Badong Formation. In the rock weathering factors, slightly weathered represented by the Qixia Formation, weakly weathered represented by the Danying Formation, strongly weathered represented by the Shilongdong Formation, and completely weathered represented by the upper part of the Jialingjiang Formation. In the rock infiltration factors, very slightly permeable represented by the four sections of the Badong Formation, slightly permeable represented by the Daye Formation, weakly permeable represented by the Penglaizhen Formation, moderately permeable represented by the Tianhepan Formation, and strongly permeable represented by the Nantuo Formation.

#### Factor correlation analysis

**Figure 6:**
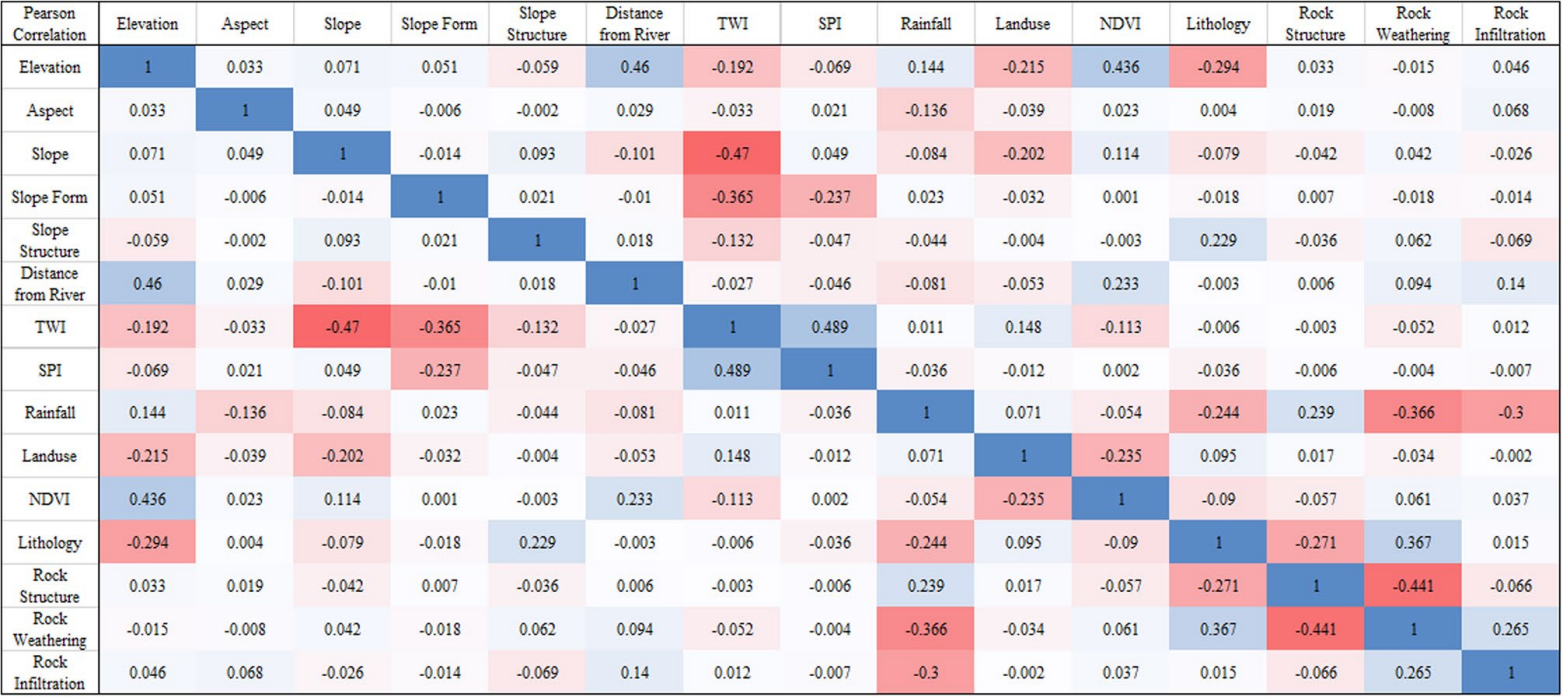
PCC matrix of 11 basic factors and 4 rock–soil characteristic factors.

To ensure the relative independence of the selected factors, IBM SPSS Statistics software is used to perform PCC analysis on the 15 factors above, to evaluate existence of strong correlation between the factors and ensure the accuracy of the LSMs. The PCC matrix between each factor is shown in Fig. 6.

It can be seen from Fig. 6 that the correlation between the factors is relatively low. The highest correlation appears between TWI and SPI, which is only 0.489, showing a weak correlation. It has no adverse effects on the establishment of the LSM model.

#### Factor multicollinearity analysis

In order to ensure that there is no multicollinearity in the selected factors in the study, which affects the calculation of the weight of the factors and causes the inaccuracy of LSM, all factors must be checked for multicollinearity. The results of multicollinearity of all factors in this study are shown in the Table 5.

**Table 5:** Multicollinearity of Factors.

Factors	Tolerance	VIF	Factors	Tolerance	VIF
Elevation	0.550	1.817	Rainfall	0.735	1.360
Aspect	0.970	1.031	Landuse	0.888	1.127
Slope	0.607	1.648	NDVI	0.771	1.297
Slope form	0.817	1.224	Lithology	0.698	1.432
Slope structure	0.919	1.088	Rock Structure	0.774	1.292
Distance from river	0.722	1.384	Rock Weathering	0.645	1.550
TWI	0.427	2.343	Rock Infiltration	0.846	1.182
SPI	0.657	1.522			

It can be seen from Table 5 that VIF values of all factors are less than 10 and TOL values are greater than 0.1, so there is no multicollinearity in the selected factors in this study.

#### Factor Relief-F analysis

**Figure 7:**
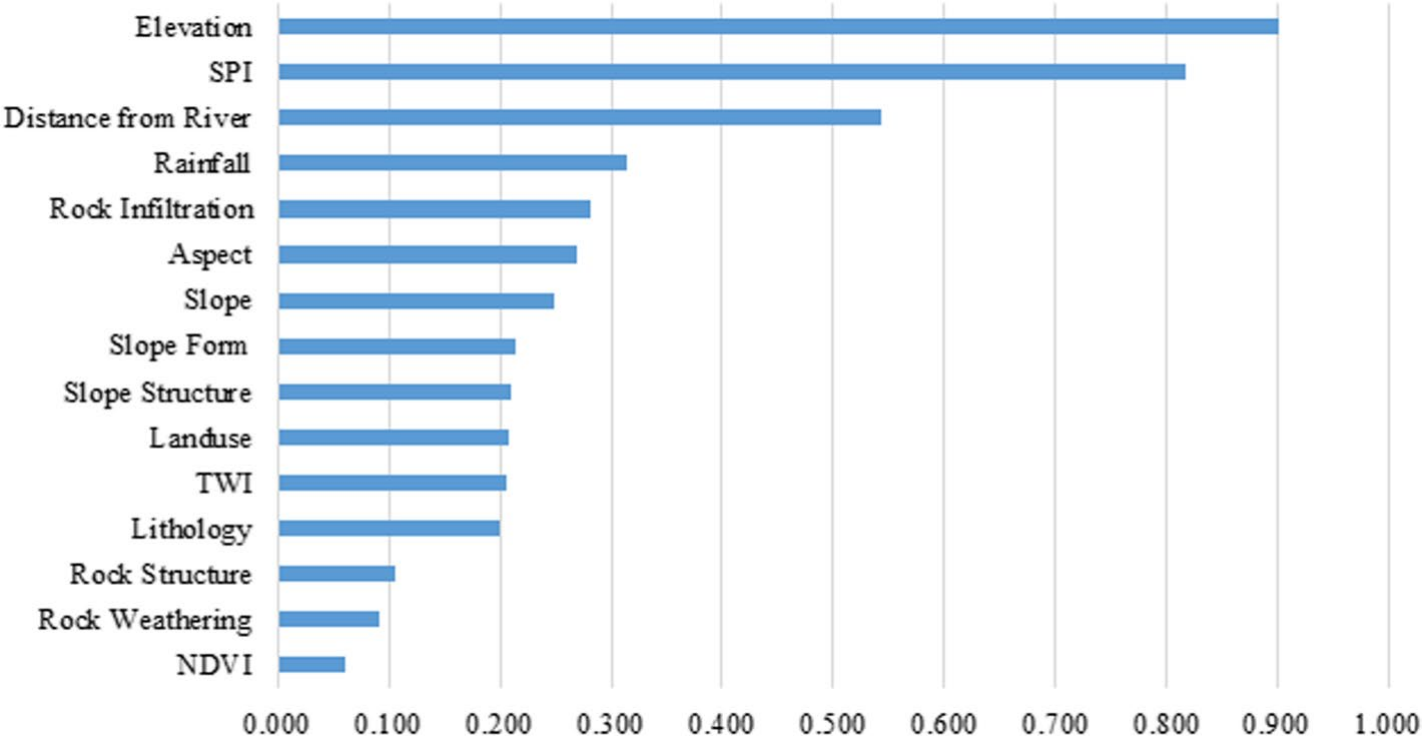
The Relief-F coefficients for 11 basic factors and 4 rock–soil characteristic factors.

Through Relief-F calculation, the factors that are not important to the occurrence of landslide events can be eliminated from the selected factors, the number of input factors for modeling can be reduced, the redundancy of model calculations can be eliminated, and the accuracy of LSM can be improved. The Relief-F coefficients of each LSM factor are shown in Fig. 7.

As shown in Fig. 7, although the Relief-F coefficients of some LSM factors are very low, for example, NDVI factor is only 0.06, but the coefficients of all factors are greater than 0, so all LSM factors are retained.

#### The final LSM factors

**Figure 8:**
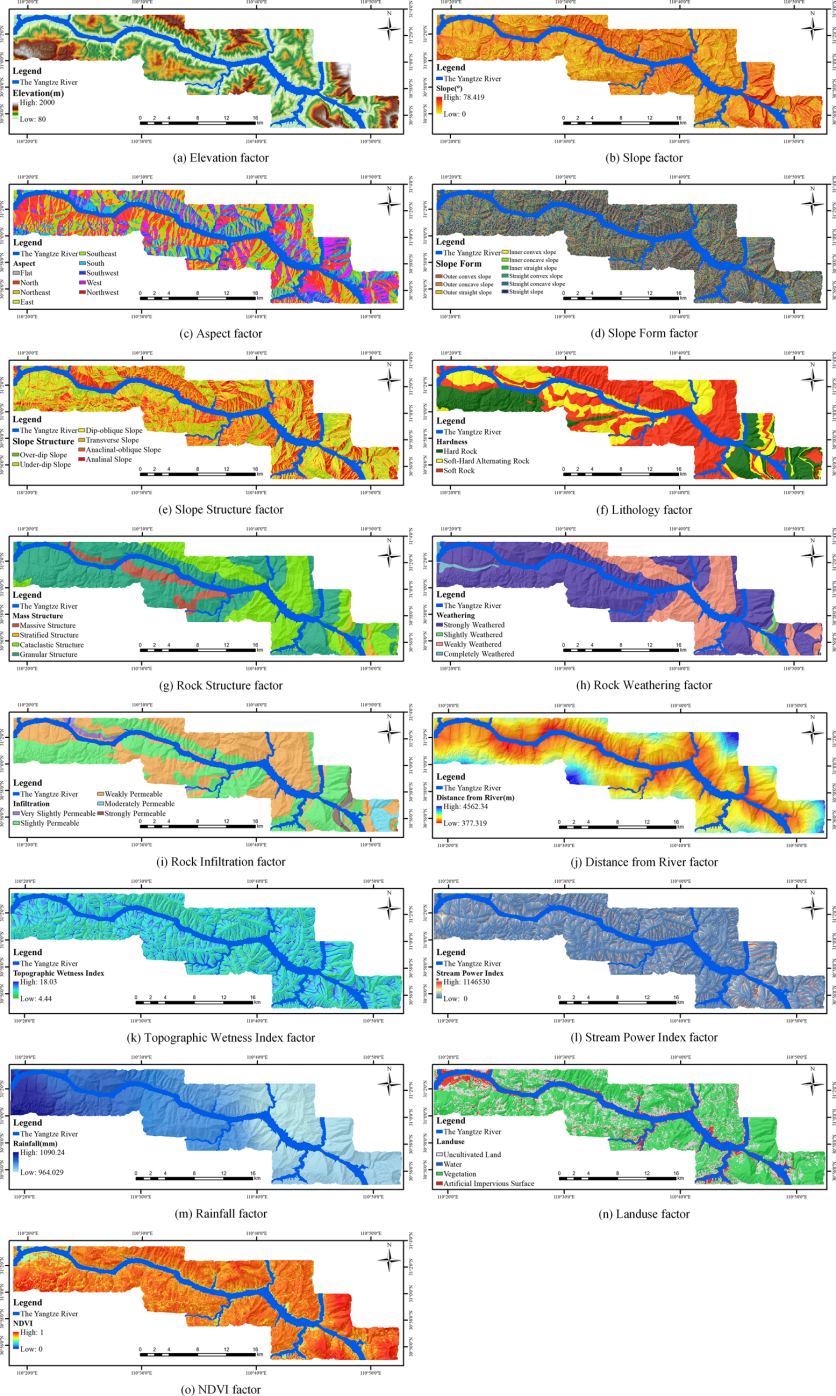
LSM factors in the study area (Drawn with ArcGIS 10.8 software, and the URL is: https://www.esri.com/en-us/arcgis/about-arcgis/overview).

The LSM factors in the study area are finally obtained after selection and analysis, as shown in Table 6 and Fig. 8.

**Table 6:** The Finally LSM Factors.

Category	Subcategory	Factor	Unit	Range
Controlling factor	Topography	Elevation	m	80.00–2000.00
		Slope		0.00–78.42
		Aspect		(1) Flat, (2) North, (3) Northeast, (4) East, (5) Southeast, (6) South, (7) Southwest, (8) West, (9) Northwest
		Slope form	–	(1) Outer convex slope, (2) Outer concave slope, (3) Outer straight slope, (4) Inner convex slope, (5) Inner concave slope, (6) Inner straight slope, (7) Straight convex slope, (8) Straight concave slope, (9) Straight slope
	Geology	Slope structures	–	(1) Over-dip slope, (2) Under-dip slope, (3) Dip-oblique slope, (4) Transverse slope, (5) Anaclinal-oblique slope, (6) Anaclinal slope
		lithology	–	(1) Hard rock, (2) Soft-hard alternation rock, (3) Soft rock
		Rock structure	-	(1) Massive structure, (2) Stratified structure, (3) Cataclastic structure, (4) Granular structure
		Rock weathering	–	(1) Slightly weathered, (2) Weakly weathered, (3) Strongly weathered, (4) Completely weathered
		Rock infiltration	–	(1) Very slightly permeable, (2) slightly permeable, (3) weakly permeable, (4) moderately permeable, (5) strongly permeable
	Hydrological	Distance from river	m	377.32–4562.34
		Topographic Wetness Index	–	4.44 ~ 18.03
		Stream Power Index	–	0–1,146,530
Influencing factor	Atmospheric precipitation	Rainfall	mm	964.029–1090.24
	Human engineering activities	Landuse	–	(1) Uncultivated land, (2) water, (3) vegetation, (4) artificial impervious surface
	Vegetation Index	Normalized Difference Vegetation Index	–	0.00–1.00

### LSM model based on three classifiers and rock–soil characteristic factors

**Figure 9:**
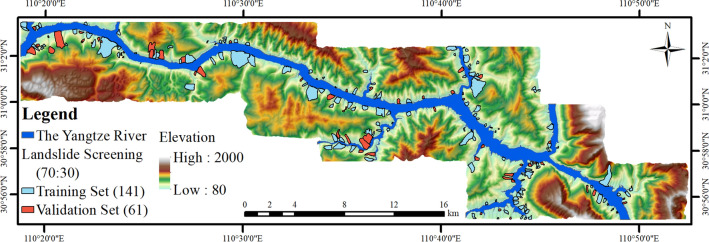
Random division result of landslides sample set in study area.

The establishment process of the LSM model based on three classifiers and rock–soil characteristic factors is as follows:

1. After calculation units and LSM factors are selected, it is necessary to establish the training sample set and the validation sample set of the LSM model. In this paper, the landslides in the study area are randomly divided according to the traditional ratio of 70:30. In other words, 141 landslides are used as the landslide distribution data in the training samples, and the remaining 61 landslides are only existed as validation samples, as shown in Fig. 9.
2. After the landslides are randomly divided, there are 141 landslides with 19,077 calculation units (the remaining 61 landslides had 14,796 calculation units) that are distributed in study area. Moreover, a buffer with 3 times raster resolution (90 m) is set up for these 141 landslides to solve the problem of landslide distribution data deviations due to inaccurate landslide surface depictions. Then, the landslides distribution data is marked as “1”, namely, landslide; the area outside the buffer area and within the study area is marked as “0”, namely, non-landslide. All the landslide calculation units are selected, and the same number of non-landslide calculation units as landslide calculation units are randomly selected, forming training sample points composed of 38,154 calculation units, to participate in the training and modeling of the LR model, the ANN model, and the SVM model.
3. Eleven basic factors including Elevation, Slope, Aspect, Slope Form, Slope Structures, Distance from River, TWI, SPI, Rainfall, Landuse, and NDVI, as well as Lithology, which is the commonly used rock–soil characteristic factor, are selected to form a combination of traditional factors. The values of these factors are assigned into the training sample points established in the previous step to obtain the training sample set, and then input into the three models for modeling.
4. All the calculation units (422,242) are used as the total sample set. All calculation units, except for the landslide training sample set involved in the modeling, were used as validation data set (403,165). Based on the model built in the previous step, the membership degree of each calculation unit to the landslide is obtained through calculations, which refers to the probability of a landslide occurring in each calculation unit. Consequently, the LSM based on traditional factor combinations is obtained.
5. As the LSM model is sensitive to the input factors, only the Lithology factor in traditional factor combinations is replaced with Rock Structure factor, Rock Infiltration factor, and Rock Weathering factor, in order to further discuss the influence of different rock–soil characteristic factors on the LSMs. Then, in a classifier model, three sample sets of LSM models are established based on different rock–soil characteristic factor combinations, generating three groups of LSMs for comparison and analysis with traditional factor combinations.
6. Repeat step (5) with different classifiers to obtain LSMs based on different classifiers and different sample sets, so as to study the influence of rock–soil characteristic factors on LSM and the stability and universality of this influence.
7. The four rock–soil characteristic factors are classified into two categories, that is, Intrinsic attribute factors and External participation factors, to generate the LSMs by three classifiers, in order to further study the influence of the introduction of different rock–soil characteristic factors on the LSM.

**Figure 10:**
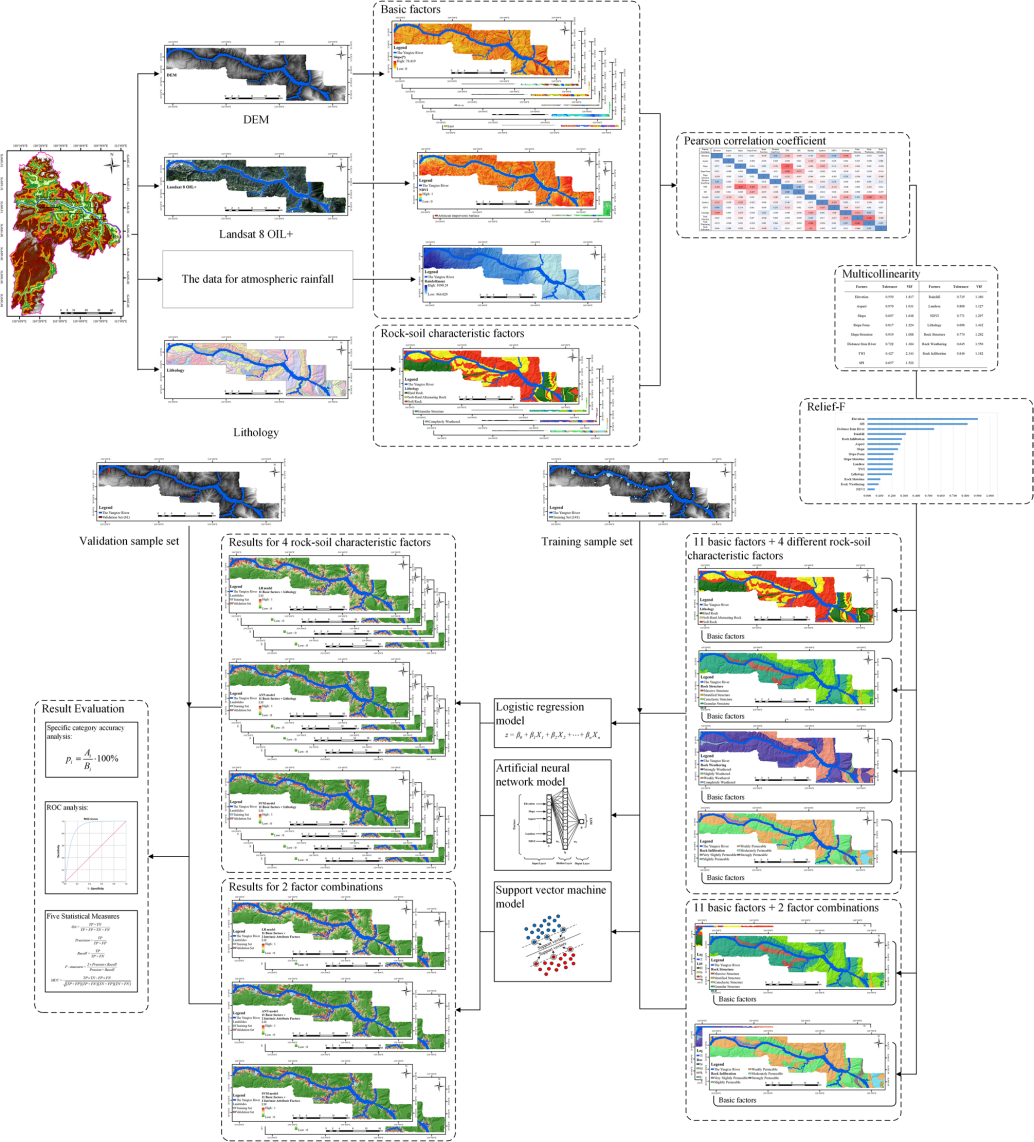
Flow chart of this paper.

The flow chart is shown in Fig. 10.

## Results and analysis

### Experimental results of traditional LSI based on lithology

**Figure 11:**
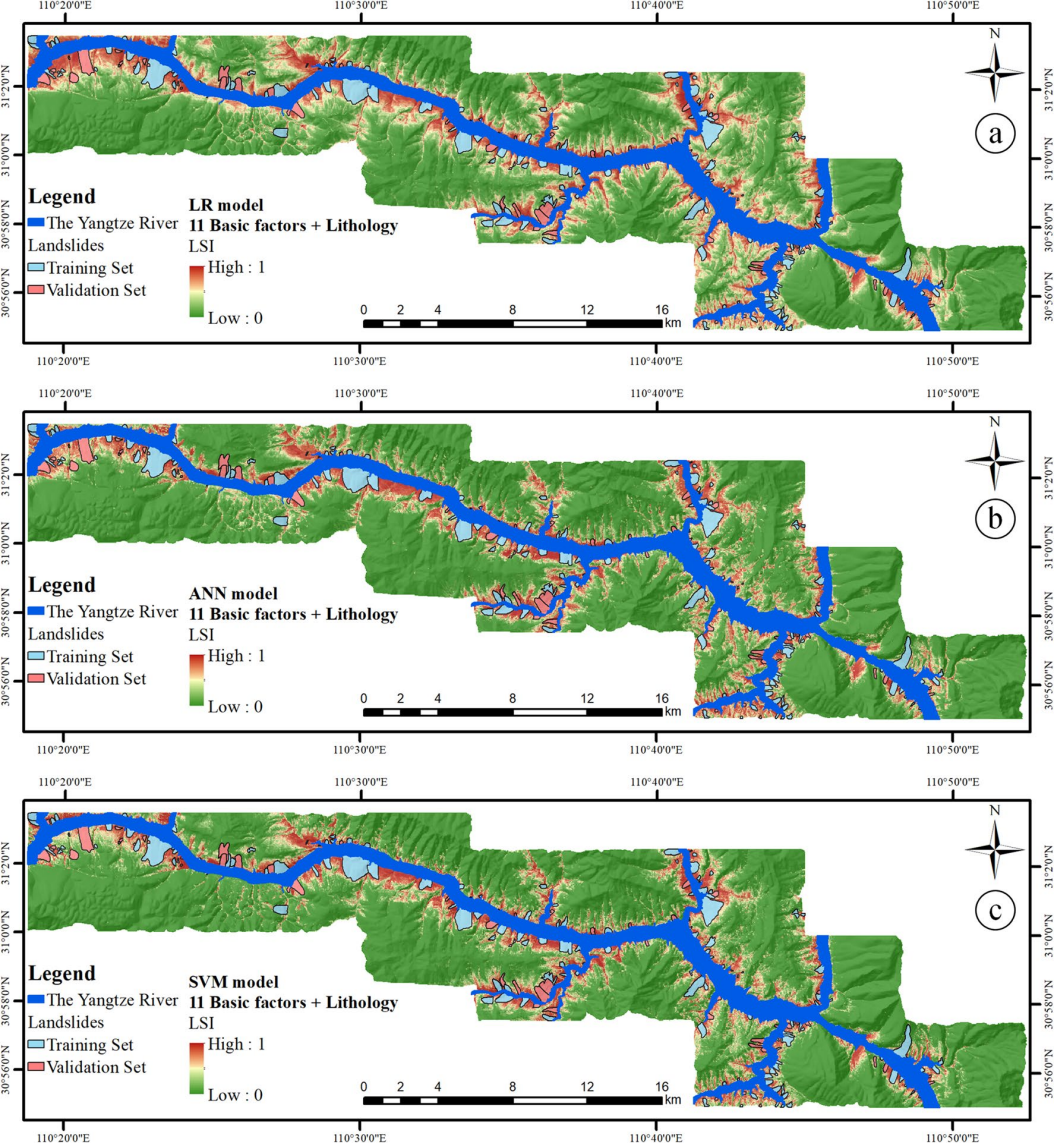
LSI based on the basic factors and lithology factor by (**a**) LR model, (**b**) ANN model, and (**c**) SVM model.

After LSM factors analysis and three classifiers modeling, the LSM model is established, and the traditional LSMs based on Lithology factor are finally obtained. The LSI is a continuous variable used to express the LSM. Its value ranges from 0 to 1. The larger the value, the greater the occurrence probability of landslides, and vice versa, as shown in Fig. 11.

### Experimental results of LSI based on the other three rock–soil characteristic factors

**Figure 12:**
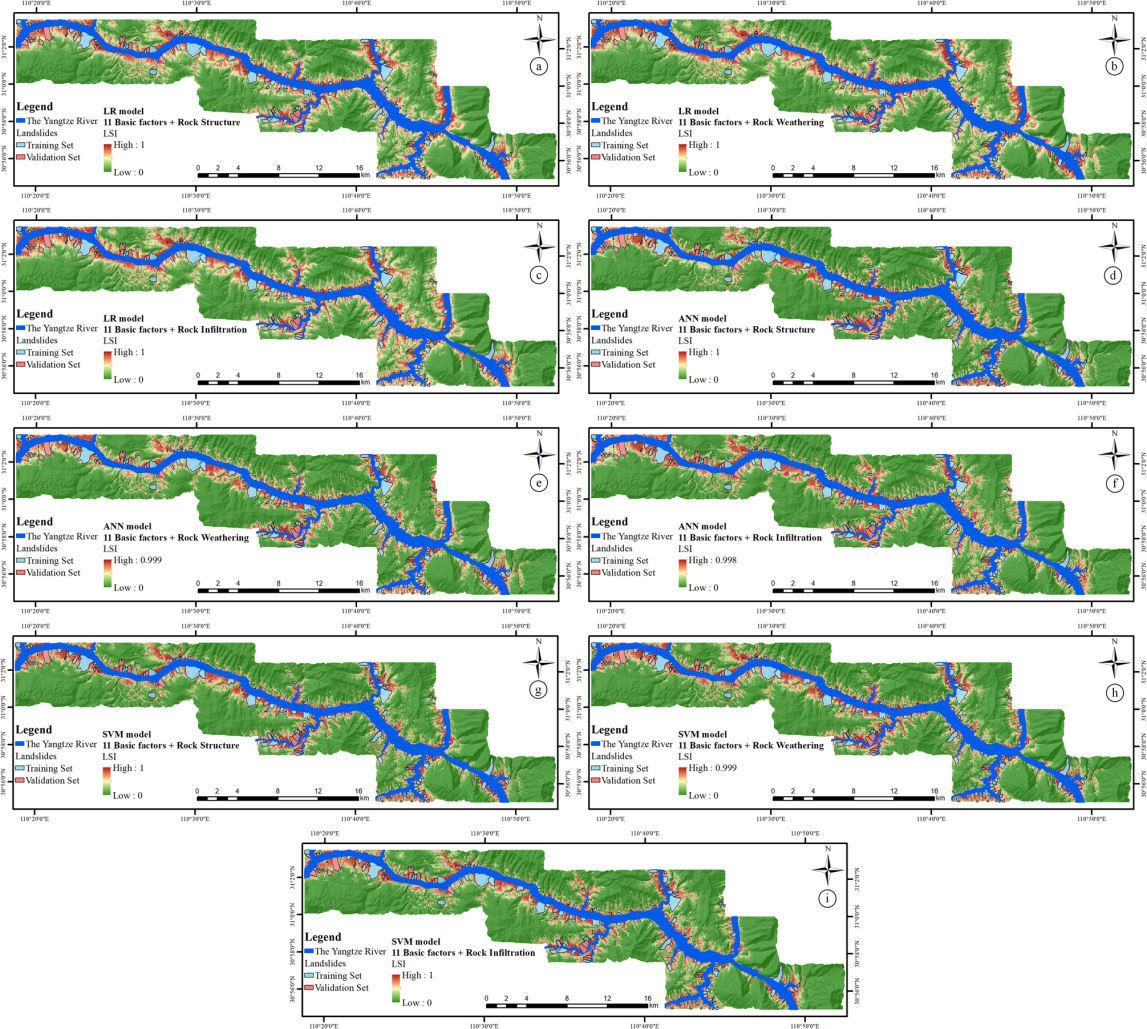
LSI based on the 11 basic factors and (**a**) rock structure factor by LR model, (**b**) rock weathering factor by LR model, (**c**) rock infiltration factor by LR model, (**d**) rock structure factor by ANN model, (**e**) rock weathering factor by ANN model, (**f**) rock infiltration factor by ANN model, (**g**) rock structure factor by SVM model, (**h**) rock weathering factor by SVM model, (**i**) rock infiltration factor by SVM model.

As mentioned in Step (5) in “LSM model based on three classifiers and rock–soil characteristic factors” section, in order to better study the influence of different rock–soil characteristic factors on the LSM, based on the principle of single variate with other conditions remaining unchanged, only the Lithology factor in traditional factor combinations is replaced with Rock Structure factor, Rock Infiltration factor, and Rock Weathering factor. Modeling with three classifiers, the nine LSIs are shown in Fig. 12.

### Experimental results of LSZ based on four rock–soil characteristic factors

**Figure 13:**
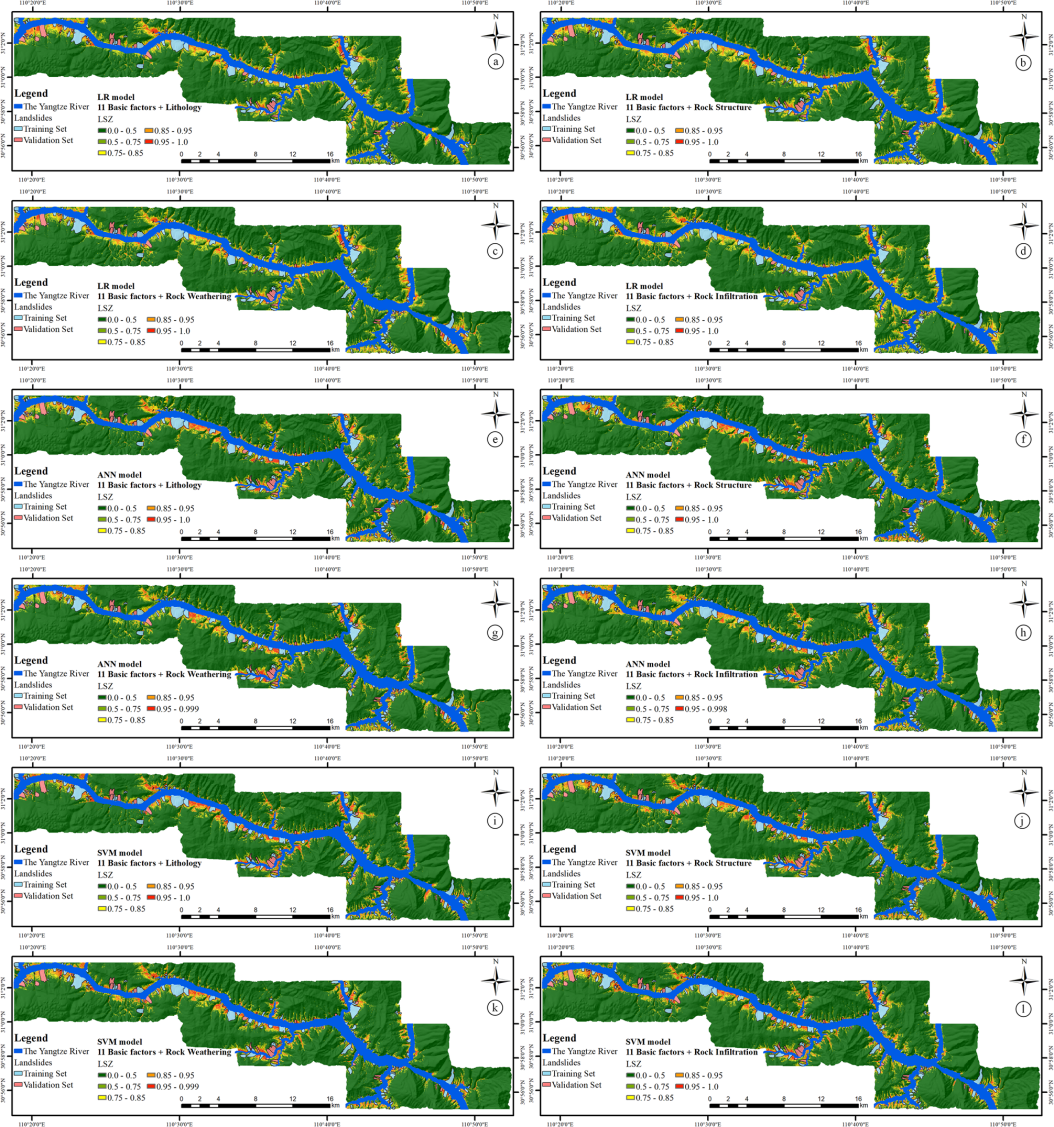
LSZs based on the 11 basic factors and (**a**) lithology factor by LR model, (**b**) rock structure factor by LR model, (**c**) rock weathering factor by LR model, (**d**) rock infiltration factor by LR model, (**e**) lithology factor by LR model, (**f**) rock structure factor by ANN model, (**g**) rock weathering factor by ANN model, (**h**) rock infiltration factor by ANN model, (**i**) lithology factor by LR model, (**j**) rock structure factor by SVM model, (**k**) rock weathering factor by SVM model, and (**l**) rock infiltration factor by SVM model.

To increase the readability of the LSIs, areas are reclassified into 5 categories in this paper—very low susceptibility, low susceptibility, medium susceptibility, high susceptibility, and very high susceptibility, according to the value range of 0–0.5, 0.5–0.75, 0.75–0.85, 0.85–0.95, and 0.95–1, respectively. The LSZs based on four different rock–soil characteristic factors are obtained, as shown in Fig. 13.

### Experimental results analysis

To quantitatively analyze the LSMs obtained in “Experimental results of LSZ based on four rock–soil characteristic factors” section, the evaluation methods mentioned in “Classifiers” section are used. It should be noted that in the analysis of the three different sample sets, including the overall sample set, the training sample set, and the validation sample set, the definitions of landslide and non-landslide are not the same, therefore, the results of these three sample sets are not comparable.

#### Specific category precision analysis

The specific category precision analysis of the LSMs based on different rock–soil characteristic factors and different classifiers is shown in Table 7.

**Table 7:** Specific category precision analysis.

Sample set	Category of susceptibility	Lithology factor (%)	Rock structure factor (%)	Rock weathering factor (%)	Rock infiltration factor (%)
**LR model**					
Overall	Very low	2.01	1.94	1.94	1.99
	Low	16.94	17.48	17.87	17.58
	Medium	28.32	27.97	27.78	27.62
	High	37.60	37.45	37.30	36.08
	Very high	46.44	47.19	45.40	42.25
Training	Very low	0.69	0.64	0.63	0.66
	Low	9.14	9.64	10.09	9.83
	Medium	18.63	18.79	18.47	18.24
	High	29.02	28.54	28.47	28.26
	Very high	40.98	41.24	39.40	35.74
Validation	Very low	1.34	1.32	1.33	1.36
	Low	9.37	9.51	9.54	9.44
	Medium	14.25	13.56	13.65	13.69
	High	16.23	16.62	16.45	14.57
	Very high	14.72	16.09	15.35	14.92
**ANN model**					
Overall	Very low	1.72	1.79	1.79	1.92
	Low	18.54	18.18	19.88	18.41
	Medium	27.52	29.00	27.75	26.90
	High	38.69	40.75	39.99	38.30
	Very high	56.17	56.01	53.87	54.95
Training	Very low	0.40	0.40	0.45	0.48
	Low	9.29	8.70	9.96	9.33
	Medium	17.69	18.74	17.84	17.29
	High	29.42	32.21	31.70	30.41
	Very high	49.55	49.68	48.37	49.46
Validation	Very low	1.33	1.40	1.35	1.46
	Low	11.13	11.27	12.10	10.93
	Medium	14.15	15.09	14.31	13.71
	High	17.63	17.53	16.83	15.53
	Very high	23.04	22.24	18.77	19.44
**SVM model**					
Overall	Very low	1.61	1.66	1.73	1.75
	Low	17.89	17.32	17.63	16.61
	Medium	32.72	28.79	32.65	32.43
	High	42.98	43.27	39.55	40.32
	Very high	50.13	51.87	50.95	50.55
Training	Very low	0.36	0.39	0.42	0.40
	Low	7.52	7.21	8.01	7.26
	Medium	22.79	16.59	22.18	22.76
	High	33.42	34.66	30.22	31.29
	Very high	43.41	45.54	45.04	44.32
Validation	Very low	1.26	1.27	1.32	1.36
	Low	12.01	11.65	11.26	10.78
	Medium	16.05	17.04	16.65	15.62
	High	20.11	18.85	18.12	18.05
	Very high	19.23	19.44	17.98	18.47

It can be seen from Table 7 that for the very high susceptibility area, the results of traditional methods based on lithological factors are not all the best in the specific category accuracy analysis of three different sample sets and three different classifiers. It ranked second in the overall and training sample sets (46.44%, 40.98%), and third in the validation sample set (14.72%) in the LR model, following result based the rock structure factor (47.19%, 41.24%, and 16.09%), which slightly better than the result based on the rock weathering factor and rock infiltration factor. The results are similar in the SVM model, the best results are based on rock structure factor (51.87%, 45.54%, and 19.44%), followed by the rock weathering factor (50.95%, 45.04%), rock infiltration factor (50.55%, 44.32%) and lithology factor (50.13%, 43.41%) in the overall and training sample sets, respectively, and followed by the lithology factor (19.23%), rock infiltration factor (18.47%), and rock weathering factor (17.98%) in the validation sample set, respectively. However, the results in the ANN model are different. In the ANN model, the top ranking factor in the training sample set is the rock structure factor (49.68%), followed by the lithology factor (49.55%), rock infiltration factor (49.46%), and rock weathering (48.37%), which is similar to the results of other models, but in the overall and validation sample sets, the top ranking factor is the lithology factor (56.17%, 23.04%), followed by rock structure (56.01%, 22.24%), rock infiltration (54.94%, 19.44%) and rock weathering (53.87%, 18.77%), respectively.

#### ROC curve and AUC value

**Figure 14:**
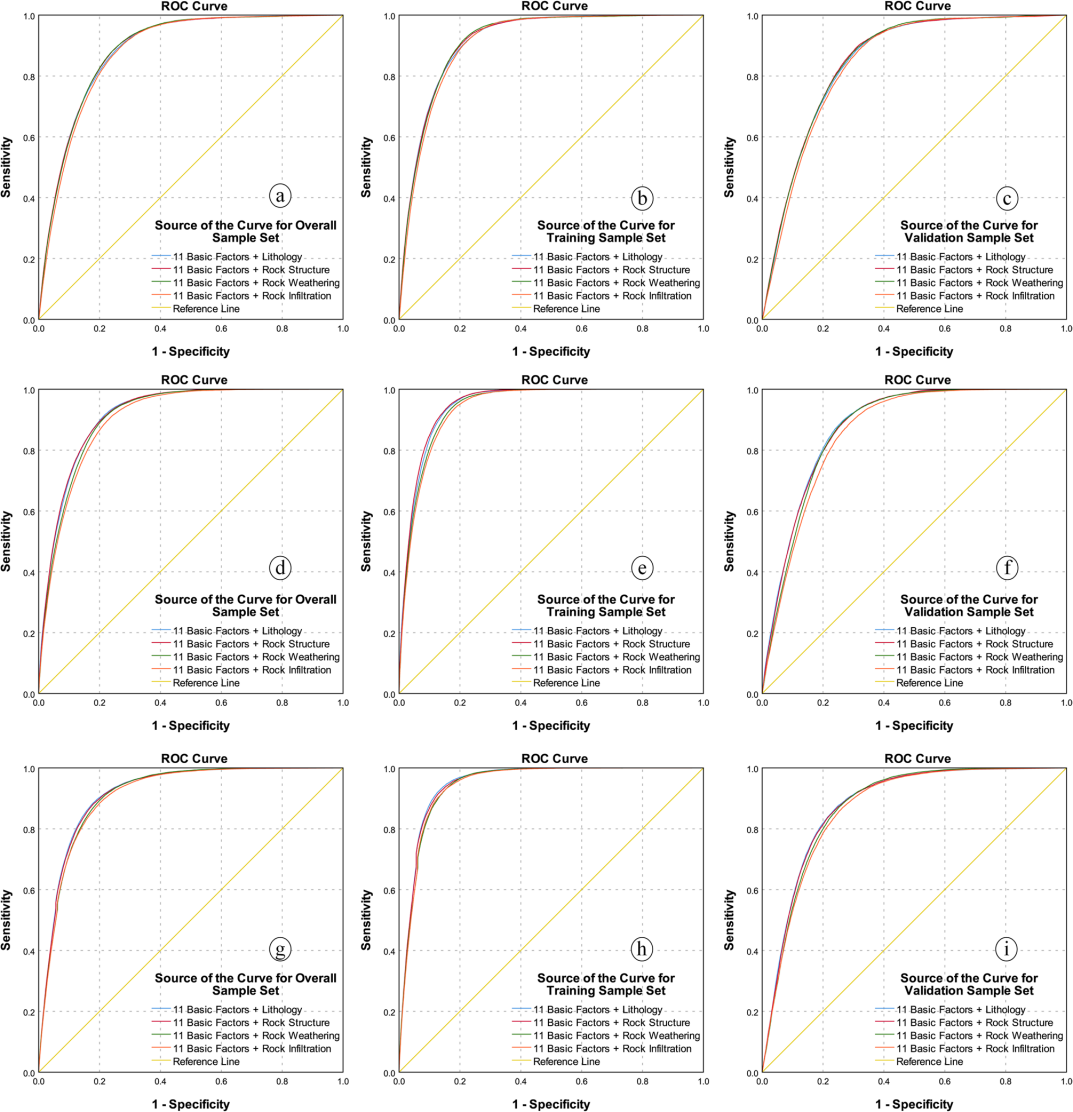
ROC curves analysis for (**a**) overall sample set by LR model, (**b**) training sample set by LR model, (**c**) validation sample set by LR model, (**d**) overall sample set by ANN model, (**e**) training sample set by ANN model, (**f**) validation sample set by ANN model, (**g**) overall sample set by SVM model, (**h**) training sample set by SVM model, and (**i**) validation sample set by SVM model.

The ROC curve of the LSMs based on different rock–soil characteristic factors and different classifiers, is shown in Fig. 14.

In the ROC curve, the curve closer to the upper left corner indicates higher precision. It can be seen from Fig. 14 that in the ROC curve analysis of the three different sample sets and three different classifiers, the rock infiltration factor results are the worst. In specifically, in the LR model, that is, Fig. 14a–c, the ROC curves based on rock structure factor and rock weathering factor are closest to the upper left corner, while the ROC curve based on rock lithology is slightly further. In the ANN model, that is, Fig. 14d–f, the ROC curve based on lithology factor and rock structure are closer to the upper left corner, while the ROC curve based on rock weathering factor further. In the SVM model, that is, Fig. 14g–i, the result is similar to the ANN model.

To better understand the results of the ROC curve analysis, AUC is used to quantitatively analyze the ROC curve, as shown in Table 8.

**Table 8:** The AUC value.

Sample set	Test result variable(s)	Area	SE^a^	Asymptotic sig.^b^	Asymptotic 95% confidence interval	
					Lower bound	Upper bound
**LR model**						
Overall	11 basic factors + lithology	0.886	0.001	0.000	0.884	0.887
	11 basic factors + rock structure	0.887	0.001	0.000	0.885	0.888
	11 basic factors + rock weathering	0.887	0.001	0.000	0.885	0.888
	11 basic factors + rock infiltration	0.880	0.001	0.000	0.879	0.882
Training	11 basic factors + lithology	0.915	0.001	0.000	0.913	0.916
	11 basic factors + rock structure	0.914	0.001	0.000	0.913	0.916
	11 basic factors + rock weathering	0.914	0.001	0.000	0.913	0.916
	11 basic factors + rock infiltration	0.909	0.001	0.000	0.908	0.911
Validation	11 basic factors + lithology	0.848	0.001	0.000	0.846	0.851
	11 basic factors + rock structure	0.851	0.001	0.000	0.848	0.853
	11 basic factors + rock weathering	0.851	0.001	0.000	0.848	0.853
	11 basic factors + rock infiltration	0.843	0.001	0.000	0.841	0.845
**ANN model**						
Overall	11 basic factors + lithology	0.917	0.001	0.000	0.916	0.918
	11 basic factors + rock structure	0.918	0.001	0.000	0.916	0.919
	11 basic factors + rock weathering	0.911	0.001	0.000	0.910	0.912
	11 basic factors + rock infiltration	0.904	0.001	0.000	0.903	0.906
Training	11 basic factors + lithology	0.947	0.000	0.000	0.946	0.948
	11 basic factors + rock structure	0.949	0.000	0.000	0.948	0.950
	11 basic factors + rock weathering	0.942	0.001	0.000	0.941	0.943
	11 basic factors + rock infiltration	0.938	0.001	0.000	0.937	0.939
Validation	11 basic factors + lithology	0.879	0.001	0.000	0.877	0.881
	11 basic factors + rock structure	0.877	0.001	0.000	0.875	0.879
	11 basic factors + rock weathering	0.872	0.001	0.000	0.870	0.874
	11 basic factors + rock infiltration	0.861	0.001	0.000	0.859	0.863
**SVM model**						
Overall	11 basic factors + lithology	0.918	0.001	0.000	0.916	0.919
	11 basic factors + rock structure	0.917	0.001	0.000	0.916	0.918
	11 basic factors + rock weathering	0.914	0.001	0.000	0.912	0.915
	11 basic factors + rock infiltration	0.911	0.001	0.000	0.909	0.912
Training	11 basic factors + lithology	0.949	0.000	0.000	0.948	0.950
	11 basic factors + rock structure	0.948	0.000	0.000	0.947	0.949
	11 basic factors + rock weathering	0.945	0.001	0.000	0.944	0.946
	11 basic factors + rock infiltration	0.944	0.001	0.000	0.943	0.945
Validation	11 basic factors + lithology	0.877	0.001	0.000	0.875	0.879
	11 basic factors + rock structure	0.877	0.001	0.000	0.875	0.879
	11 basic factors + rock weathering	0.873	0.001	0.000	0.871	0.875
	11 basic factors + rock infiltration	0.868	0.001	0.000	0.866	0.870

^a^Under the nonparametric assumption.

^b^Null hypothesis: true area = 0.5.

The conclusion of Table 8 and Fig. 14 is consistent, and have more details than ROC curve analysis. As show in Fig. 14, in the Table 8, the performance of rock infiltration factor in different sample sets and different classifiers is the worst. For the LR model, the AUC values based on rock structure factor and rock weathering factor are the same (0.887, 0.851), slightly better than the AUC values based on lithology factor (0.886, 0.848) in overall and validation sample sets, and in the training sample set, the AUC value based on lithology factor is the best (0.915), slightly better than the AUC values based on rock structure factor and rock weathering factor (0.914). For the ANN model, the AUC values based on rock structure factor (0.918, 0.949) are better than the AUC values based on the lithology factor (0.917, 0.947) and rock weathering factor (0.911, 0.872) in the overall and training sample sets, however, the AUC value based on the lithology factor (0.879) is the best, followed by the AUC values based on the rock structure factor (0.877) and rock weathering factor (0.872) in the validation sample set. For the SVM model, the AUC values based on the lithology factor is the best in three sample sets (0.918, 0.949, and 0.877), and the AUC values base on rock structure factor (0.917, 0.948, and 0.877) is almost equal to them, which is better than the AUC value based on rock weathering factor (0.914, 0.945, and 0.876).

#### Five statistical methods

In order to analyze the statistical results of LSM results obtained with different sample sets and different classifiers, the calculation results of five statistical methods, including OA, precision, recall, F-measure, and MCC are shown in Table 9. In this section, only the overall sample set is used.

**Table 9:** The results of five statistical methods.

Statistical methods	Lithology factor	Rock structure factor	Rock weathering factor	Rock infiltration factor
**LR model**				
OA	80.36%	80.49%	80.60%	79.73%
Precision	0.2643	0.2666	0.2678	0.2582
Recall	0.8122	0.8179	0.8181	0.8148
F-measure	0.3988	0.4022	0.4035	0.3921
MCC	0.4343	0.4371	0.4383	0.4287
**ANN model**				
OA	84.51%	84.82%	83.91%	83.10%
Precision	0.3205	0.3242	0.3107	0.2976
Recall	0.8309	0.8225	0.8248	0.8131
F-measure	0.4626	0.4651	0.4513	0.4357
MCC	0.4881	0.4898	0.4784	0.4650
**SVM model**				
OA	85.51%	85.28%	84.68%	84.33%
Precision	0.3377	0.3334	0.3229	0.3174
Recall	0.8391	0.8354	0.8295	0.8280
F-measure	0.4815	0.4766	0.4649	0.4589
MCC	0.5043	0.5000	0.4900	0.4849

The Table 9 shows that, in the LR model, the results of statistical calculation based on rock structure factor are the best (80.49%, 0.2666, 0.8179, 0.4022, and 0.4371), followed by the result based on rock weathering factor (80.60%, 0.2678, 0.8181, 0.4035, and 0.4383), lithology factor (80.36%, 0.2643, 0.8122, 0.3988, and 0.4343), and rock infiltration factor (79.73%, 0.2582, 0.8148, 0.3921, and 0.4287). In the ANN model, the results are slightly different. The results of statistical calculation based on rock structure factor are still the best (84.82%, 0.3242, 0.8225, 0.4651, and 0.4898), but the second and third ranking has changed. The results based on lithology factor ranked second (84.51%, 0.3205, 0.8309, 0.4626, and 0.4881), rock weathering factor ranked third (83.91%, 0.3107, 0.8248, 0.4513, and 0.4784), and rock infiltration ranked last (83.10%, 0.2976, 0.8131, 0.4357, and 0.4650). Compared with the other two models, the results have changed a lot in the SVM model. The results of statistical calculation based on lithology factor are the best (85.51%, 0.3377, 0.8391, 0.4815, and 0.5043), followed by the results based on rock structure factor (85.28%, 0.3334, 0.8354, 0.4766, and 0.5000), the rock weathering factor (84.68%, 0.3229, 0.8295, 0.4649. and 0.4900), and rock infiltration factor (84.33%, 0.3174, 0.8280, 0.4589, and 0.4849).

#### Summary of experimental results based on 4 rock–soil characteristic factors

In the specific category accuracy analysis, a total of nine results were obtained from three sample sets and three classifiers. Among these nine results in the very high susceptibility category, the best ones are the results based on rock structure factor and the lithology factor (7 and 2 times, respectively), and the second ranked results are those based on lithology factor, rock weathering factor and rock structure factor (4, 3 and 2 times, respectively). In this analysis, it can be found that the results based on rock structure did not appear in the third and fourth place, that is, its influence on LSM will be more stable, while the traditional lithology factor, which appeared in the first, second and fourth place, indicates the instability of its influence on LSM.

In the AUC value, which also obtains nine results from three sample sets and three classifiers, due to the occurrence of the same AUC value, the results based on rock structure factor, lithology factor and rock weathering factor are ranked first (5 times, 5 times and 2 times, respectively), and the second ranked results based on rock structure factor, lithology factor and rock weathering factor (7 times, 3 times and 3 times, respectively). In this analysis, it can be observed that although the number of occurrences of rock structure factor and lithology factor are the same in the first place of the results, just like the specific categories accuracy analysis, the results based on rock structure factor are more concentrated and ranked higher than those of lithology factor, indicating that rock structure factors have a greater influence on LSM.

Among the five statistical methods, the study only analyzed the overall sample set, therefore, only three results were obtained. The results show that the top ranking is the result based on rock structure factor and lithology factor (2 times and 1 time, respectively), which also indicates statistically that the rock structure factor is more important than the lithology factor in the LSM.

In summary, although in some cases, the results based on rock structure factors are not the best, and often alternate with the result based on lithology factors in the first place, but in most cases, the results based on rock structure factors are the best. It shows that its stability in LSM and its influence on LSM are better than lithological factors.

### Experimental results and analysis based on two rock–soil characteristic factor combinations

**Figure 15:**
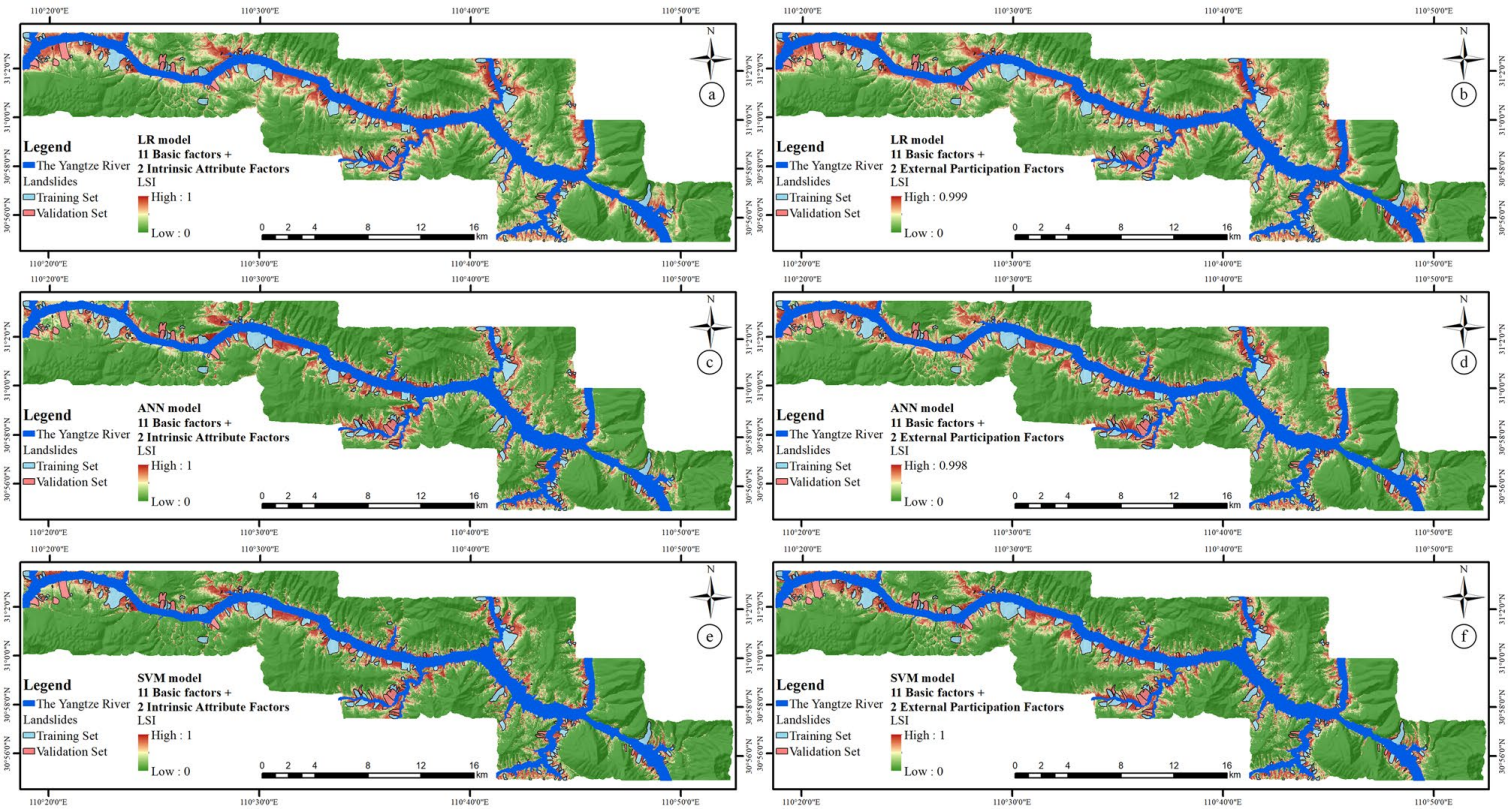
LSI based on the 11 basic factors and (**a**) intrinsic attribute factors by LR model, (**b**) external participation factors by LR model, (**c**) intrinsic attribute factors by ANN model, (**d**) external participation factors by ANN model, (**e**) intrinsic attribute factors by SVM model, and (**f**) external participation factors by SVM model.

To further study the influence of the introduction of different rock–soil characteristic factors on the LSM, the four rock–soil characteristic factors are classified into two categories— “Intrinsic attribute factors” and “External participation factors”. According to Table 4, Lithology factor and Rock Structure factor are the internal attributes of rock soil, and are determined by the nature of rock–soil mass, while Rock Infiltration factor and Rock Weathering factor can be realized with the participation of external conditions (water, wind, sunlight, air, and so on). Based on this, two new factor combinations can be obtained, namely, the “Basic factors and intrinsic attribute factors” and “Basic factors and external participation factors”. According to the new factor combinations and three classifiers, the LSI based on the intrinsic attribute factor and the external participation factor can be obtained, as shown in Fig. 15.

**Figure 16:**
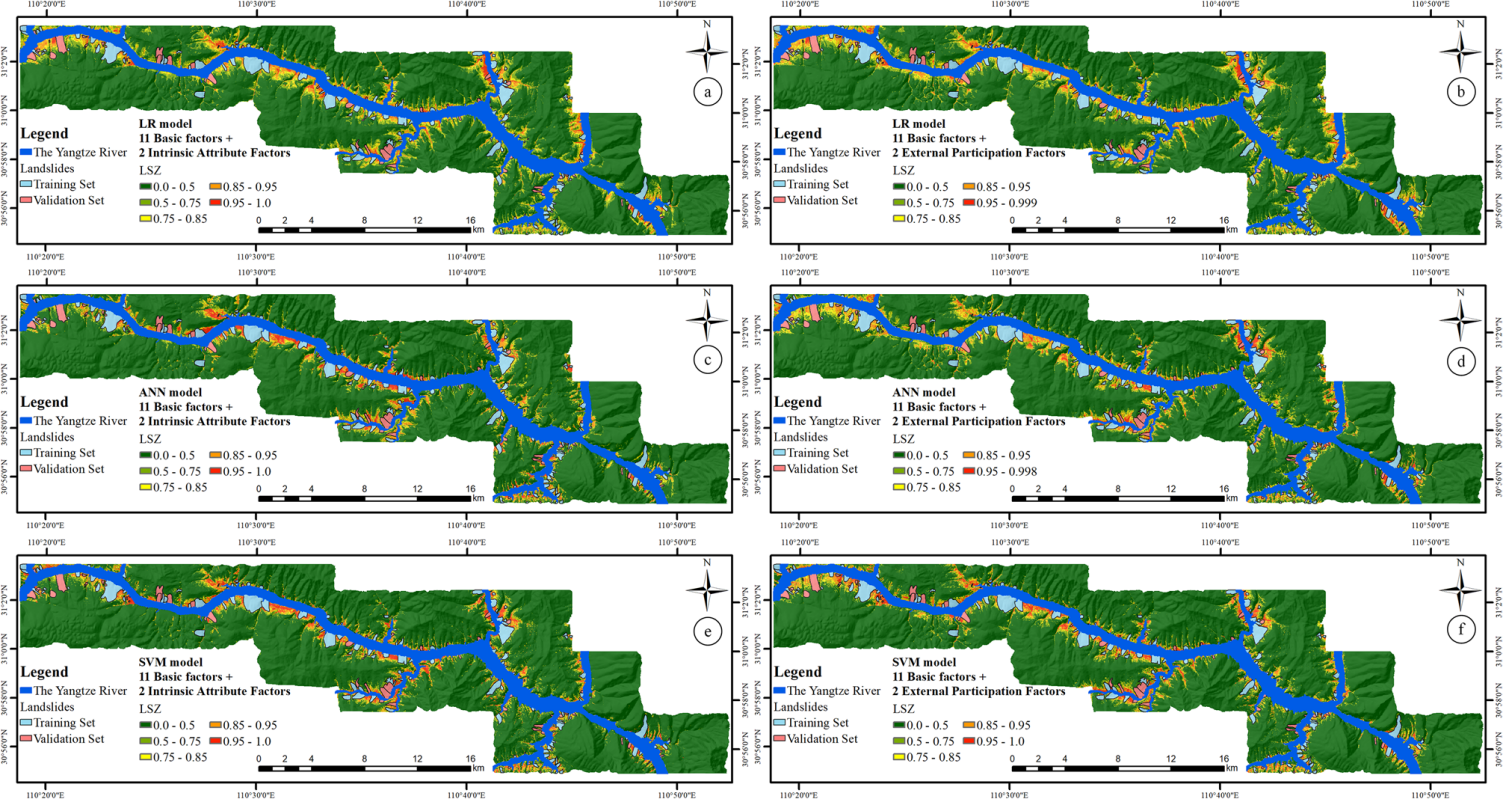
LSZ based on the 11 basic factors and (**a**) intrinsic attribute factors by LR model, (**b**) external participation factors by LR model, (**c**) intrinsic attribute factors by ANN model, (**d**) external participation factors by ANN model, (**e**) intrinsic attribute factors by SVM model, and (**f**) external participation factors by SVM model.

Similarly, to better interpret the LSIs, the results are reclassified with the interval of 0–0.5, 0.5–0.75, 0.75–0.85, 0.85–0.95, and 0.95–1.0, and the LSZs are obtained, as shown in Fig. 16.

**Figure 17:**
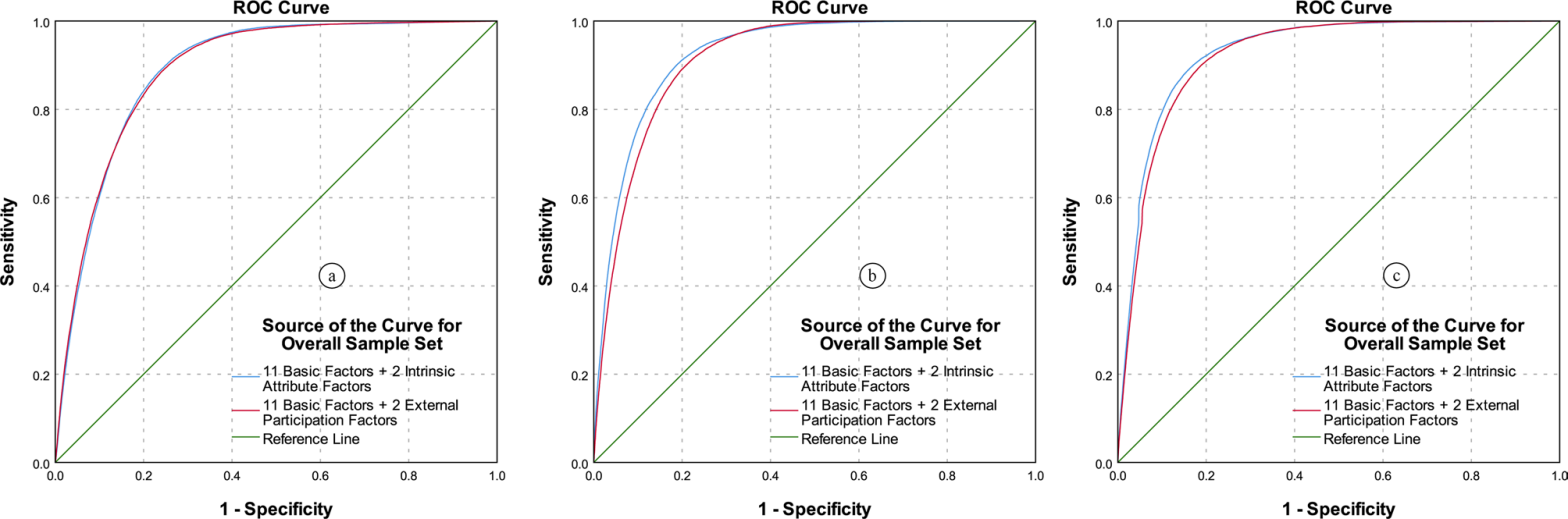
The ROC curves analysis for intrinsic attribute factors and external participation factors by (**a**) LR model, (**b**) ANN model, and (**c**) SVM model.

To analyze the two groups of LSMs based on different types of rock–soil characteristic factor combinations, the two evaluation methods and five statistical methods mentioned in the “Result evaluation model” section are still adopted in this section. The results are shown in Fig. 17 and Table 10.

**Table 10:** The result of different evaluation methods based on Intrinsic attribute factors and External participation factors.

Category of susceptibility	Very low (%)	Low (%)	Medium (%)	High (%)	Very high (%)
**Specific category precision analysis for overall sample set**					
LR model					
11 basic factors + intrinsic attribute factors	1.92	17.22	28.11	38.69	48.90
11 basic factors + external participation factors	1.83	18.42	28.24	37.79	45.85
ANN model					
11 basic factors + intrinsic attribute factors	1.76	18.68	29.54	42.85	57.99
11 basic factors + external participation factors	1.75	18.77	28.86	40.49	53.56
SVM model					
11 basic factors + intrinsic attribute factors	1.55	18.40	28.80	47.41	54.29
11 basic factors + external participation factors	1.64	17.54	28.83	44.73	51.95

Test result variable(s)	Area	SE^a^	Asymptotic sig.^b^	Asymptotic 95% confidence interval	
				Lower bound	Lower bound
**AUC value for overall sample set**					
LR model					
11 basic factors + intrinsic attribute factors	0.891	0.001	0.000	0.890	0.892
11 basic factors + external participation factors	0.891	0.001	0.000	0.890	0.893
ANN model					
11 basic factors + Intrinsic attribute factors	0.926	0.001	0.000	0.925	0.927
11 basic factors + external participation factors	0.915	0.001	0.000	0.913	0.916
SVM model					
11 basic factors + intrinsic attribute factors	0.929	0.001	0.000	0.928	0.930
11 basic factors + external participation factors	0.922	0.001	0.000	0.921	0.923

Statistical methods	OA	Precision	Recall	F-measure	MCC
**Five statistical methods for overall sample set**					
LR model					
11 basic factors + intrinsic attribute factors	81.12%	0.2750	0.8274	0.4128	0.4462
11 basic factors + external participation factors	81.02%	0.2726	0.8186	0.4090	0.4429
ANN model					
11 basic factors + intrinsic attribute factors	86.58%	0.3548	0.8217	0.4956	0.5153
11 basic factors + external participation factors	84.27%	0.3164	0.8284	0.4579	0.4841
SVM model					
11 basic factors + intrinsic attribute factors	87.48%	0.3751	0.8417	0.5189	0.5360
11 basic factors + external participation factors	85.88%	0.3437	0.8361	0.4872	0.5089

^a^Under the nonparametric assumption.

^b^Null hypothesis: true area = 0.5.

From a qualitative perspective, it can be seen from Fig. 17 that the ROC curve of the LSM based on the “Intrinsic attribute factors” is closer to the upper left corner than the ROC curve of the LSM based on the “External participation factors”, indicating that its prediction effect is better.

From a quantitative perspective, as can be seen in Table 10, whether it is specific category precision analysis, ROC curve analysis, or five statistical methods, the LSM based on intrinsic attribute factors is better than that based on external participation factors in three classifiers. It shows that the intrinsic attribute factor has a more important role and influence on LSM than the external participation factors.

A comprehensive analysis of the data in Tables 7, 8, 9 and 10 reveal some interesting phenomena. Compared to the results based on rock structure factors and traditional lithology factor, the LSM based on intrinsic attribute factors has a significant improvement in the evaluation method used in the study. There is no doubt that the improvement of the evaluation results is due to the fact that the intrinsic attribute factors can express rock–soil characteristics more comprehensively, but it also needs to be noted that it may also be due to more factors participating in the LSM model during the modeling process.

In view of the latter possible problem, it can be seen from the data in Tables 7, 8, 9 and 10 that the LSM based on external participation factors is not all better than the results when the four rock–soil characteristics factors are combined with the basic factors alone; for example, in the overall sample set of the LR model, the LSM based on external participation factors are only slightly better than those based on rock permeability and rock weathering, and it even gives the worst results in the overall sample set of the ANN model.

It shows that the improvement of the LSM is not only due to the increase in the number of factors participating in the LSM modeling, but it is more closely related to the significance of the factors participating in the modeling of the development and occurrence of landslides.

## Discussions

LSM plays a vital role in the management and prevention of landslide disasters. Therefore, it is very important to improve the accuracy of prediction and help managers and decision makers obtain more accurate LSI and LSZ^45^. To this end, this study expands the lithology factor, which is traditionally considered as the only factor representing rock–soil characteristics among geological factors. According to the national standards of the People’s Republic of China^65,66^, three new rock–soil characteristic factors have been obtained: rock structure, rock weathering and rock infiltration, and using the traditional LR model, ANN model and SVM model to obtain different LSMs.

In general, morphological, geological, and hydrological conditions are highly correlated with landslide occurrence^6,45^. In this study, PCC coefficients, multicollinearity and Relief-F methods were used for factor screening and examination to ensure the validity of LSM factors. Elevation was found to be the most critical LSM factor when using the Relief-F method because it determines the stress distribution on slopes and is associated with human activities that affect landslide stability. These observations are consistent with previous studies^45,68^.

Once the LSM factor is determined, the basic factors can be combined with the rock–soil characteristic factors to obtain LSM based on different rock–soil characteristic factors by constructing different sample sets and using different classifiers. Two evaluation methods (specific category accuracy analysis, ROC curve analysis and AUC value) and five statistical methods (OA, Precision, Recall, F-measure, MCC) are used to evaluate LSM results. The experimental results show that the traditional method simply considered lithology as the only rock–soil characteristic factor, which is one-sided or even wrong. In most cases, the results based on the rock structure factor are better than those based on lithology factor. From all the experiments, the former ranked more highly and concentrated, indicating that rock structure factor has more influence on the LSM, and this influence is more stable.

To further verify the influence of rock–soil characteristic factors on LSMs, the four rock–soil characteristic factors in the study were classified into internal attribute factors (lithology factor and rock structure factor) and external participation factors (rock weathering factor and rock infiltration factor) according to whether external conditions were involved as classification standard to formed two factor combinations with the basic factors, respectively, and then three classifiers are used to obtain LSMs based on the overall sample set. The experimental results show that the results based on internal attribute factors are better than those based on external participation factors in all evaluation methods. That is to say, in terms of rock–soil characteristics, the influence of internal attributes of the rick–soil mass themselves on LSMs are greater than that with the participation of external conditions. This can be seen very implicitly in previous studies for factor analysis^12,32,67^.

In recent years, researchers have paid attention to the importance of LSM factors for LSM. Some researchers have found some special LSM factors^23^, some are committed to studying the LSM factors in different regions^24–26,28^, some are concerned about the influence of different factor combinations on LSMs^27^, some are attention about the influence of the mathematical attributes of the factors themselves on LSM^31^, and some are more concerned about the number of LSM factors^6^. However, little attention has been paid to the influence of rock–soil characteristic factors in LSM. The rock–soil characteristic factors play an important role in the occurrence of landslide, which are very important in geological factors and one of the most essential controlling factors for the development of landslides. Therefore, it is very promising to explore the influence of rock–soil characteristic factors on LSM.

Although in the study, the rock–soil characteristic factors, especially the rock structure factor and the inherent attribute factor composed of the rock structure factor and lithology factor, have achieved good performance in LSM, but there are two points that need to be noted. First, in the national standards of the People's Republic of China, there are many other rock–soil characteristic factors. Whether these factors can be used in LSM research and what kind of influence they have on LSM is worth exploring. Second, researchers all know that in different study areas, influenced by topography, geology, hydrology, meteorology rain, earthquakes, human engineering activities and other factors, each LSM factors often exhibits different importance. Whether rock–soil characteristic factors perform well in other study areas is also the focus of further research.

## Conclusion

In traditional LSM studies, researchers tend to include lithology as the only geological factor related to rock–soil characteristic in LSM modeling. Rocks can be classified into hard rocks, soft rocks, and soft-hard interbedded rocks according to their lithology or engineering geological conditions. For a more detailed study, rocks can be further classified into 5–7 or more categories by combining the hardness and proportions of rocks in different strata. However, it is undeniable that regardless of the classification method used, the concept of geotechnical properties is only related to rock hardness. This is a one-sided understanding of the concept of rock–soil characteristic and the influencing factors.

In this study, the application of rock–soil characteristic factors in LSM is researched using the section from Zigui to Badong in the TGRA as the study area. This method, which is based on the stratigraphic properties of the study area, is universal and can be applied to other regions in the world with similar characteristics. All 15 LSM factors used in this study passed the validity checks of PCC coefficients, multicollinearity, and Refile-F methods. Different LSMs were obtained using traditional LR models, ANN models and SVM models based on different combinations of 11 basic factors and 4 geotechnical characteristics factors. The validation of the results was carried out with objective indicators from two evaluation methods and five statistical methods.

The experiments confirmed the following conclusions. First, the proposed rock structure factor and internal attribute factors are more practical for landslide management and prevention than the traditional lithology-based LSM due to the improved accuracy. Second, the results based on the rock structure factor ranked high and concentration in most of the evaluations. Third, the internal attribute factors consisting of rock structure and lithology had the best results in all evaluations. And finally, the evaluation results show that the rock structure factor and internal attribute factors have a stronger and more stable influence on LSM. In summary, LSM factor analysis, especially the rock–soil characteristics factor, is promising for landslide spatial prediction. In the future, our research will investigate more efficient factor analyses for LSM.

## Data Availability

The public data such as remote sensing data and DEM data, can be downloaded directly through the link provided in Table 1. However, basic geographic data, basic geological data, and landslide distribution data are all confidential data in China. According to the requirements of relevant laws, these confidential data have been decrypted when we use them. Any researchers in related fields that need these decrypted data can contact the corresponding author to obtain them.
